# The Renaissance of Cyclin Dependent Kinase Inhibitors

**DOI:** 10.3390/cancers14020293

**Published:** 2022-01-07

**Authors:** Tobias Ettl, Daniela Schulz, Richard Josef Bauer

**Affiliations:** 1 Department of Oral and Maxillofacial Surgery, University Hospital Regensburg, 93053 Regensburg, Germany; daniela.schulz@ukr.de; 2 Center for Medical Biotechnology, Department of Oral and Maxillofacial Surgery, University Hospital Regensburg, 93053 Regensburg, Germany

**Keywords:** PD-L1, HNSCC, CDK, CDKI, cell cycle inhibition/blockade, palbociclib, flavopiridol, ribociclib, seliciclib, abemaciclib, trilaciclib, CDK4/CDK6, chemosensitization, radiosensitization, immunosensitization, synergy

## Abstract

**Simple Summary:**

This review provides an overview of the state of knowledge and general understanding of CDK inhibitors currently under development or clinically approved, with a particular focus on the treatment of head and neck cancer. Especially in combination therapy, cyclin-dependent kinase inhibitors exhibit a synergistic effect by attenuating chemo-, radio-, or immune-resistance in some tumor entities, thus improving the success of cancer therapy.

**Abstract:**

Cyclin-dependent kinases (CDK) regulate cell cycle progression. During tumor development, altered expression and availability of CDKs strongly contribute to impaired cell proliferation, a hallmark of cancer. In recent years, targeted inhibition of CDKs has shown considerable therapeutic benefit in a variety of tumor entities. Their success is reflected in clinical approvals of specific CDK4/6 inhibitors for breast cancer. This review provides a detailed insight into the molecular mechanisms of CDKs as well as a general overview of CDK inhibition. It also summarizes the latest research approaches and current advances in the treatment of head and neck cancer with CDK inhibitors. Instead of monotherapies, combination therapies with CDK inhibitors may especially provide promising results in tumor therapy. Indeed, recent studies have shown a synergistic effect of CDK inhibition together with chemo- and radio- and immunotherapy in cancer treatment to overcome tumor evasion, which may lead to a renaissance of CDK inhibitors.

## 1. Introduction

One fundamental aspect of cancer is uncontrolled cell growth. When a cancer increases in size, this process reflects an increase in the number of cells due to the process of cell division. This feature is such an important characteristic of cancer cells that a sustained cell proliferation was nominated as one of the six original hallmarks of cancer by Hanahan and Weinberg in 2000 [1].

In order to inhibit tumor proliferation, understanding the mechanisms that control the division process of cancer cells is required. The generation of identical daughter cells is a highly regulated process in healthy cells. However, alterations that may occur in malignant cells can finally lead to uncontrolled cell proliferation. For a better understanding of the mode of action of cyclin dependent kinase inhibitors (CDKIs), we first describe the cell cycle in general and how cyclins and cyclin-dependent kinases (CDKs) play an important role during cell cycle progression. There are studies designed to stop the cell division process in cancer and therefore CDKIs have been developed. However, the complexity of cellular regulation mechanisms makes specific inhibition of proliferation in tumor cells very challenging. In this review we present the progress that has been made in the development of CDKIs so far, as well as the challenges and limitations of CDKI treatment that come along in cancer therapy. Despite the many disappointing drawbacks in the history of CDKI development that we will discuss in detail, hormone receptor positive metastatic breast cancer is one of the tumor entities for which there are already clinically approved CDKIs by the FDA that are an integral part of cancer treatment [2].

With CDK inhibition, there are some challenging aspects to deal with. In the context of CDK inhibition was the lack of inhibitor specificity. Furthermore, inhibition of CDKs may not alter just a single but a variety of processes in the cell. Depending on the tumor entity, specific mutations, as well as the differentiation status, tumor cells do not always respond identically and predictable to CDK inhibition. After all, inhibition of CDKs may not only affect tumor cells, but also other cell types like fibroblasts and immune cells. Finally, also questions arise regarding proper dosing and the balance of efficacy and adverse outcomes, which may vary depending on the CDK inhibitor type.

As we discuss in this review, interestingly, CDKIs have not necessarily performed as well as monotherapy. Instead, CKDIs have been shown to be particularly effective in combination with chemo-, radio-, or immunotherapy. Such promising combination therapy strategies are of great interest for tumor entities such as head and neck cancer (HNSCC), upon which we will particularly focus in this review. In HNSCC, for instance, the 5-year overall survival rate has hardly improved for decades, despite all medical progress in the last years [3]. The combination of CDKIs with the simultaneous inhibition of mutated signaling pathways used by tumor cells as an evasion mechanism as well as the simultaneous stimulation of the immune system seem to be promising strategies for future tumor therapies which, in our opinion, could lead to a renaissance of CDKIs. 

## 2. Cyclin-Dependent Kinases

It was only about two decades ago that the key concepts of CDK biology were discovered by studying yeast and the synchronous cycles of division in embryo extracts. Not much later Leland H. Hartwell, R. Timothy Hunt and Paul M. Nurse were awarded the Nobel Prize for their achievements [4]. Over time, further studies revealed the distinct functions of CDKs. The evolutionary expansion of the CDK family in mammals led to the classification of CDKs into three cell-cycle-related (CDK1, CDK4 and CDK5) and five transcriptional subfamilies (CDK7, CDK8, CDK9, CDK11 and CDK20). However, there are many other important CDKs that transmit and respond to a wide variety of growth regulatory signals and therefore influencing cell proliferation. For example, CDK4 and CDK6 ensure smooth entry into the cell cycle. CDK1 was found to be a key factor in mitosis and CDK2 to be essential in the replication of DNA in higher eukaryotes. CDK7, CDK8/19, CDK9, CDK12/13 were identified to play important roles during the regulation of RNA transcription [5,6,7]. 

Considering the tremendous role of CDKs during cell division, it is not surprising that tumor cells have developed a number of strategies to disable or bypass these key components [8]. In the last 20 years, researchers have developed numerous drugs to manipulate the activity of CDKs to inhibit uncontrolled tumor proliferation. Many of these drugs have already been used as cancer treatment, but with varying degrees of success. In the past, monotherapy with CDKIs showed rather often disappointing results. Recently, however, CDKIs have been used more effectively in therapy, especially when used as combination therapy with chemo- and radiotherapy. Combination therapy with CDKI has also achieved initial success in the context of immunotherapy, which will be discussed in more detail later in this review. Their success may now lead to a veritable renaissance of CDKIs.

### 2.1. Role and Function of CDKs

Cell division is essential for the development and maintenance of a healthy organism. Cell cycle progression is a highly regulated process. The cell cycle consists of several distinct phases. In G0, the quiescent state, the cell enters right after a successful cell division and remains before it probably enters a proliferative state. When the cell switches to a proliferative state, the cell enters G1 and the synthesis phase (S phase), where DNA synthesis occurs, followed by G2 and mitosis phase (M phase), in which the cell divides into two identical daughter cells. Cell cycle checkpoints ensure that processes are carefully executed and that proper duplication of DNA and other cell contents has occurred before the cell enters the next phase. Any mitotic cell division requires correct DNA replication to take place in S phase. During mitosis, the machinery required for chromosome division is present, leading to the formation of daughter cells. Here, CDKs exert an enormous functional influence and perform distinct functions during distinct phases of the cell cycle. Ranging from the initiation of DNA replication sites along the chromosomes before S phase, they phosphorylate histone proteins during S and M phases, resulting in chromatin conformation. Besides that, they regulate the degradation of the nuclear membrane by phosphorylation of nuclear proteins, such as lamins or nucleoporins, at the beginning of M phase and engage in phosphorylation of centrosome-associated proteins in preparation for mitosis.

Each phase transition in the cell cycle is regulated by a specific subset of cyclins and CDKs. CDKs are involved as key regulatory enzymes that sensitively control all cell cycle transitions through restriction points (R-points) [9]. Among the serine/threonine (Ser/Thr) kinases, CDKs represent a distinct subfamily of enzymes. Together with cyclins, CDKs are the core components of the molecular machinery that determines whether a cell continues to progress through the cell cycle or enters a stage where cell growth is arrested. 

In addition, there is a whole series of CDKs known to keep the transcriptional machinery running. These transcriptional CDKs (tCDKs) control the activity of RNA polymerase from binding to the promoter to recycling of the polymerase. This process, called the transcription cycle, integrates multiple sources of information to ensure that RNA synthesis at all genomic is precisely adapted to the requirements of the cell.

### 2.2. Regulation of Cell Cycle CDKs

To ensure an uninterrupted cell cycle progression, CDKs are continuously monitored. The activity of CDKs is regulated by phosphorylation and dephosphorylation of the catalytic subunit as well as by the interaction with cyclins. CDKs and cyclins form functional complexes in which cyclins activate the catalytic activity of cell phase specific CDK interaction partners. This in turn triggers phosphorylation signals of a variety of responder molecules at their Ser/Thr residues and thereby directs cells through the cell cycle.

**Figure 1:**
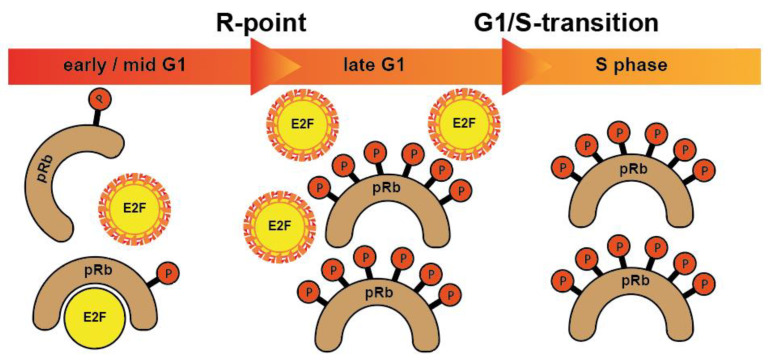
pRb proteins bind to and block E2F transcription factors, controlling the expression of E2F responsive genes in specific cell cycle phases. After being hyperphosphorylated at the restriction point (R-point), pRb proteins release E2F transcription factors allowing them to activate the transcription of numerous genes crucial for cell cycle progression. When cells enter S phase, E2Fs are degraded. Hence, E2Fs are active only from late G1 until entry into S phase.

More specifically, when cells from G0 enter the cycle, CDK4 and CDK6 form active complexes with D-type cyclins (D1, D2, D3) and, among other proteins, phosphorylate the retinoblastoma protein (pRb) [10]. Rb is one of the foremost investigated tumor suppressor protein substrates of CDK which prevents cells from entering cell cycle under normal conditions. Phosphorylation of Rb by CDKs causes its inactivation and allows cells to enter a new cell cycle. In its phosphorylated form, the binding of RB1 to E2F transcription factors is released, which is necessary for cell cycle progression. In a non- or low-phosphorylated condition, pRb forms repressor complexes by binding to E2F transcription factors. Further, the CDK2/cyclin E complex phosphorylates Rb family members and inactivates them [11]. The subsequent release of transcription factors allows the cells to pass the R-point and move from G1 to the S phase [10,12] (Figure 1). 

**Figure 2:**
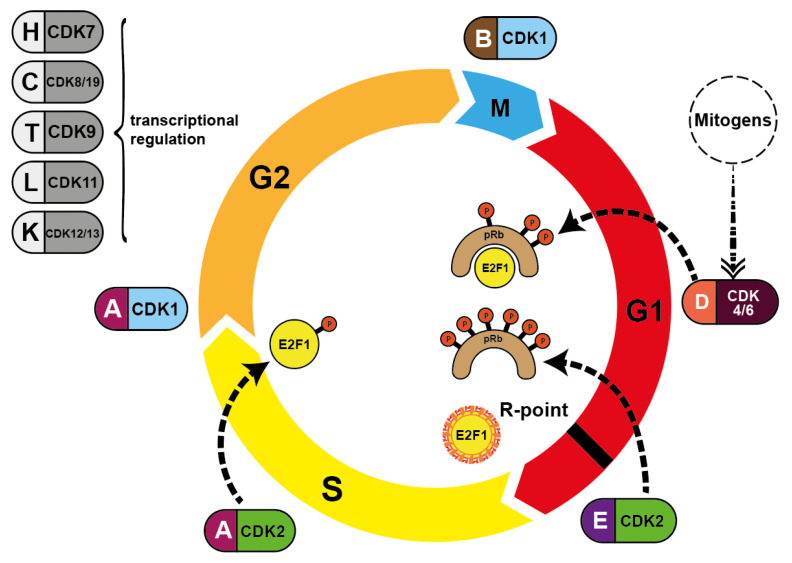
Pairing of cyclins with the respective CDK during cell cycle progression. Mitogenic stimulation leads to the synthesis of D-type cyclins via mitogenic pathways, activating CDK4/CDK6/cyclin D and ultimately CDK2/cyclin E. CDK4/CDK6 phosphorylate Rb proteins (dotted lines) and CDK2 further phosphorylates Rb subsequently reversing suppression of transcription factor E2F1. This allows DNA synthesis to occur. S phase is terminated when CDK2/cyclin A phosphorylates E2F1, blocking its DNA-binding ability. CDK7, CDK8/CDK19, CDK9 CDK11 and CDK12/CDK13 are all involved in transcriptional initiation and DNA elongation processes.

During S phase progression, CDK2 exchanges its E-cyclin partners and replaces them with cyclins A1 and A2. These cyclins in turn then switch from CDK2 to CDK1 (CDC2) in late S phase. Once the cell enters G2 phase, CDK1 binds to cyclins B1 and B2. Finally, this CDK1/cyclin B1/B2 interaction drives the passage through M phase by controlling many events from prophase to telophase [9]. Once the M phase is complete, the B-cyclin level drops again to almost zero. The level then rises again over the course of the entire cell cycle, in preparation for the next cell division. All other cyclins undergo similar cycles and induce the distinct phases of cell division (Figure 2). 

### 2.3. Regulation of Transcriptional CDKs

**Figure 3:**
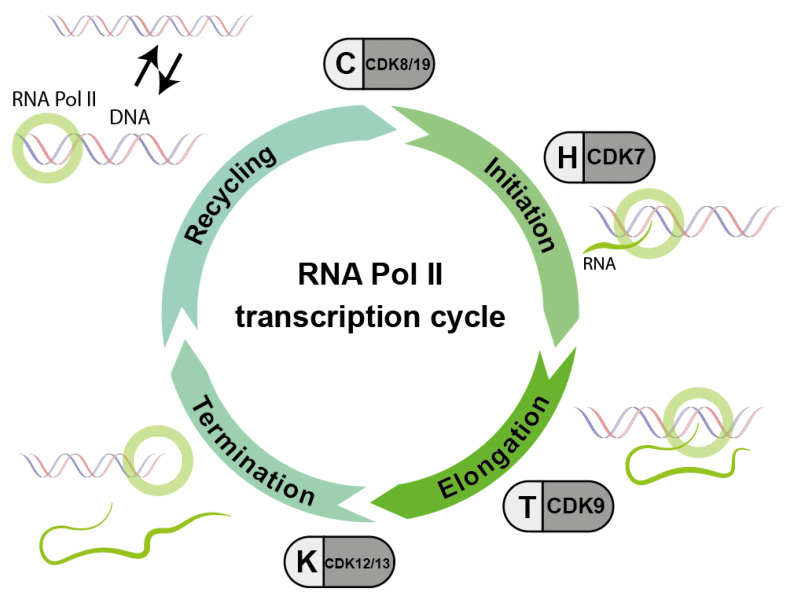
Basic scheme of the sequential action of different cyclin/CDK complexes which are important for the phosphorylation cycle and consequently the activity of RNA polymerase II (RNA Pol II). Accordingly, inhibitors against these CDKs interfere with the RNA transcription process (modified according to Parua et al. [14]).

In addition to the CDKs that regulate the cell cycle, a whole series of CDKs are known to keep the transcriptional machinery running. These transcriptional CDKs (tCDKs) control the activity of RNA polymerase from binding to the promoter to recycling of the polymerase (Figure 3). During the progression of RNA transcription, multiple CDK-cyclin complexes act sequentially to promote transitions between and progression through the different stages of transcription: first the initiation of RNA transcription, second the elongation of RNA and third the termination of RNA Polymerase II (RNA Pol II). Unphosphorylated RNA Pol II is able to re-enter the cycle, which is called RNA Pol II recycling. 

This process, called the transcription cycle, integrates multiple sources of information to ensure that RNA synthesis at all genomic loci is precisely tailored to the cell’s requirements. Our understanding of the transcription cycle is still limited. More recently, attempts have been made to manipulate transcriptional activity for cancer control through inhibition of tCDKs, both in basic and applied sciences. Targeting tCDKs may provide a sufficient therapeutic window to be effective in the clinic [13].

### 2.4. Cell Intrinsic CDK Inhibition

For cancer treatment, the goal is to specifically interrupt cell cycle progression to stop tumor growth. For this purpose, CDK inhibitors have been developed. However, the cell cycle is of course also regulated naturally. The action of CDKs is controlled by specific activation and inactivation by other proteins, cell intrinsic CDK inhibition. Typically, cyclin/CDK complexes are activated by phosphorylation of the CDK-activating kinase (CAK). On the contrary, these complexes can be negatively regulated by a number of cell intrinsic CDKIs [12]. In healthy cells, this mechanism prevents uncontrolled proliferation. In cancer cells, however, these mechanisms are disturbed. Cyclin D1, for instance, is susceptible to extrinsic mitogenic stimulation, providing a link between the external environment and the intrinsic cell cycle machinery. 

**Figure 4:**
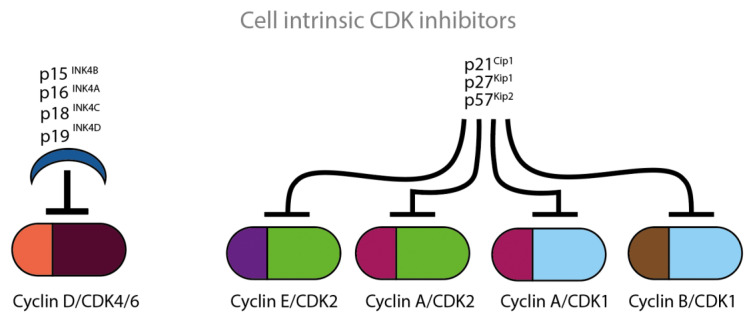
Cell intrinsic CDKCDKIs block the actions of CDKs at various time points of the cell cycle. The four illustrated INK proteins specifically inhibit cyclin D/CDK4/CDK6 complexes which are active in the G1 phase of the cell cycle. The three illustrated Cip/Kip proteins inhibit the remaining cyclin/CDK complexes which are active throughout the whole cell cycle.

Based on their structural and functional properties, naturally occurring CDKIs belong to two large families, INK4 and Cip/Kip, which are displayed in Figure 4. The cell intrinsic CDK inhibition mechanism involves interaction between the p16- and CDK6-charged binding domains, resulting in reduced kinase activity and a decreased cyclin-binding surface. 

The INK4 family includes the CDKIs p15, p16, p18 and p19 and consists of tandem repeats of an ankyrin-like sequence [15,16,17,18]. The INK4 family members inhibit the D-type cyclin activity through selective interaction with CDK4 and CDK6. The CDK interacting protein/Kinase inhibitory protein (Cip/Kip) family includes the CDKIs p21, p27 and p57 and is characterized by a homologous amino-terminal domain that contains contiguous cyclin- and CDK-binding regions [19,20,21]. Amongst others, the Cip/Kip family members inhibit the activity of CDK2 by binding both to cyclin E/CDK2 and to cyclin A/CDK2 complexes. Cip/Kip family members can also target CDK4/6 and might work either as activators or inhibitors [22,23]. P27 was first identified as cell intrinsic CDKI. P27 is known for its ability to block the activity of cyclin E/CDK2 and cyclin A/CDK2 in cells arrested in G1, by TGF-ß, lovastatin or contact inhibition [20,21] (Figure 4).

There are also numerous data in literature showing a reduction on tumor progression by cell intrinsic CDK inhibition. P16, p53, p21 and p27 proteins represent endogenous CDKIs. It was shown that induction of these inhibitors caused arrest of the cells in G1/S phase, or directly forced cells into apoptosis in various tumor cells, such as breast, prostate and lung cancer [24,25,26,27,28,29,30].

### 2.5. Dysregulation of Catalytic Components of CDKs

Dysregulation of the cell cycle may lead to the manifestation of various clinical conditions, and in the worst case, to cancer. Here, especially the dysregulation of the catalytic components of CDKs can lead to the disruption of regular cell cycle progression [8,9].

For example, mutations of CDK4 and CDK6, which have been found in melanoma and other tumor types, can disrupt the regular progression of the cell cycle [17]. Additionally, overexpression of the regulatory subunit of cyclins is common cause of dysregulation in malignancies. Amplification of cyclin D, cyclin E and cyclin B1 has been reported in leukemia, lymphoma and various solid tumors as a result of gene amplification, rearrangement or translocation [11,12,17]. CDK2 is shown to be dysfunctional in several malignancies, including lung cancer (cyclin A/CDK), melanoma, osteosarcoma and ovarian cancer (cyclin E/CDK2), which is usually due to overexpression of cyclin E and/or cyclin A [9]. 

To prevent uncontrolled progression of the cell cycle, resulting in uncontrolled proliferation and the accumulation of mutations in malignant cells, many studies have aimed to develop specific and selective CDKIs as a potential therapeutic approach to target cancer. However, due to the complex mechanisms by which the cell cycle is regulated, there have been many challenges in finding a successful tumor therapy. In the next paragraph we will discuss the possibilities of CDK inhibition and the different types of CDKIs.

## 3. Pharmacological Compounds for CDK Inhibition

In past decades, many efforts have been made to develop kinase inhibitors capable of modulating cyclin-CDK complexes, either by mimicking the function of natural CDK inhibitors such as p21, p16, and p27 or by directly modulating cyclin-CDK complexes or their targets. Retinoblastoma protein is one of the foremost investigated tumor suppressor protein substrates of CDK which prevents cells from entering cell cycle under normal conditions. Phosphorylation of Rb by CDKs causes its inactivation and allows cells to enter a new cell cycle. Many cancers are associated with hyperactivation of CDKs due to mutation of CDK genes or cell intrinsic CDK inhibitor genes. Therefore, CDK modulators are of great interest as novel therapeutics against cancer and have led to the discovery of several CDK inhibitors in the clinic. 

There is still no consensus on whether peptides or small molecules are more suitable for therapeutic applications. Peptides are more selective because they are derived from linear protein sequences. Peptides should mimic the catalytic or regulatory subunits of cell cycle control complexes, but on the other hand they usually have poorer pharmacokinetic properties. In contrast, small molecules have better pharmacokinetic properties but lower specificity. The reason is that many protein kinases have high sequence similarity in the active site. 

### 3.1. Small Molecules

Growing knowledge about protein sequences, structures and interacting partners has made it possible to optimize peptides in terms of molecular design. Due to their high target specificity, peptides offer a promising opportunity for CDK inhibition. 

The first therapeutic approaches were made with pharmacologically active peptides designed to mimic the catalytic or regulatory subunits of cell cycle control complexes. For instance, peptides based on p21 were designed to block the cyclin E/CDK2 complex. To be delivered into cells, these peptides were fused with the cargo peptide penetratin, a cell penetrating peptide (CPP) derived from the *Drosophila Antennapedia* protein (*Antp*) [31]. Depending on which region of the protein the peptides originated, the inhibition was more or less effective [32]. Some well-known small molecules are flavopiridol, roscovitine, SNS-032, R-547, AT-7519, PD-0332991, UCN-01, indirubin derivatives, dole-3 carbinol, paullones, and hymenialdisine [33]. Several of them will be described in more detail in their function and mode of action later in the text.

However, small molecules have low specificity, as many protein kinases have high sequence similarity in the active site. This would explain why small peptides cannot achieve desired treatment results. The pharmacokinetic properties of these molecules are often very poor in vivo, meaning the large and highly charged peptides are unstable and rapidly degraded and do not reach the necessary concentration at the actual tumor site [34]. 

### 3.2. Stapled Peptides

**Figure 5:**
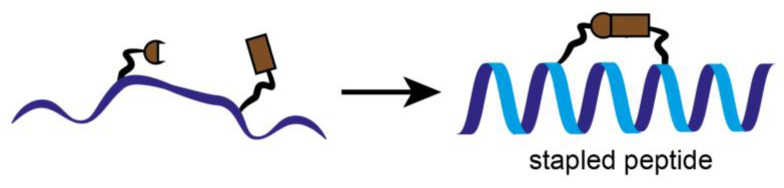
Schematic representation of stapled peptides. To keep the peptide into a more stable alpha helical form and optimize its properties, individual amino acids are crosslinked together. Stapled peptides can enter cells, bind to therapeutic targets and modulate biological structures and properties, and may be of great interest as inhibitors of protein-protein interactions (PPI).

In parallel to the classical inhibition attempts, stapled peptides were used to inhibit the interaction between CDK4 and its binding partner cyclin D1, recently. Stapled peptides are short peptides, typically arranged in an alpha-helical conformation, which is constrained by a synthetic brace, a so-called staple (Figure 5). This conformation allows an increase target affinity, increased cell penetration and protection against proteolytic degradation [35].

Stapled peptides derived from the α-helix of CDK4 involved in the primary interaction with cyclin D1, are designed to inhibit cyclin D/CDK4. The peptides are constrained with hydrocarbon staples, modified amino acids (α-methyl, α-alkenylglycine) into strategic positions of a peptide sequence which induce an alpha-helical conformation [36]. This structural property stabilizes the peptides and makes them much less prone to degradation by proteases. As a result, their ability to interact with their target and penetrate cell membranes is enhanced [37,38]. They fold into an α-helix in solution in the absence of cyclin D1, and interact with cyclin D1 with high affinity compared to the unstapled peptides [39]. Stapled peptides target the main interface between CDK4 and cyclin D which is essential for the kinase activity of CDK4. 

However, stapled peptides are not very specific since they have been shown to affect the kinase activity of various other CDKs since the amino acids involved in the α5-helix that interacts with the C helix of CDKs are highly conserved. Although an anti-proliferative effect was observed in various NSCLC cell lines, no inhibition of lung tumors in mice by stapled peptides was observed after intravenous injection. This was probably because the concentration at the tumor site was too low. In orthotopic lung tumors of mice, the peptides were able to successfully inhibit the growth only in combination with abemaciclib [40].

A well-known stapled peptide is the α-helical peptide ALRN-6924. It has entered a clinical phase I trial in patients with solid tumors and lymphomas [41]. ALRN-6924 activates p53-dependent transcription at the single-cell and single-molecule levels and exhibits biochemical and molecular on-target activity in leukemia cells in vitro and in vivo. Dual MDMX/MDM2 inhibition by ALRN-6924 inhibits cellular proliferation by inducing cell cycle arrest and apoptosis in cell lines and primary AML patient cells, including leukemic stem cell-enriched populations, and disrupts functional clonogenic and serial replication capacity. Furthermore, ALRN-6924 significantly improves survival in AML xenograft models. Our study provides mechanistic insights that support further testing of ALRN-6924 as a therapeutic approach in AML and other cancers with wild-type p53 [42].In breast cancer, letrozole or fulvestrant are used in addition to stapled peptides. For MM cisplatin, paclitaxel, bortezomib or dexamethasone are applied in combination [43,44,45]. 

### 3.3. PROTACs and Molecular Glue

**Figure 6:**
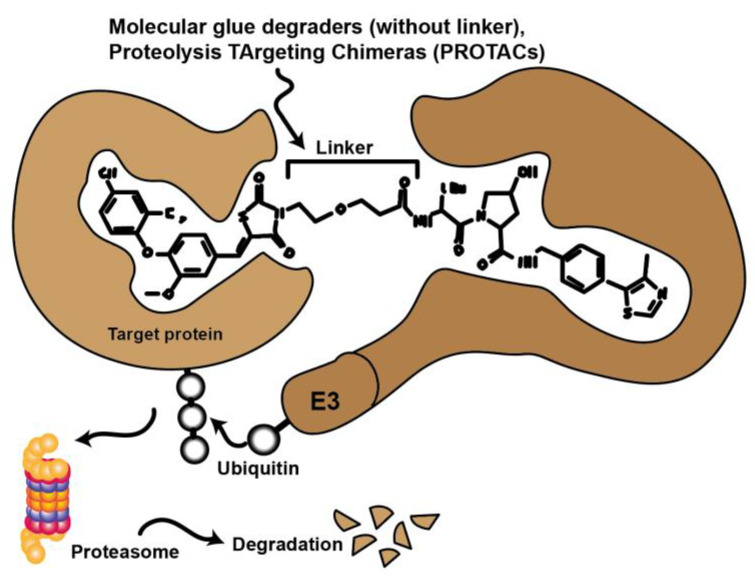
Schematic representation illustrating the mode of action of proteolysis targeting chimeras (PROTACs) and molecular glues. Chemical compounds that bind to both target protein and ubiquitinylating E3 ligase to bring these proteins into spatial proximity. The target protein is then tagged and proteasomally degraded.

Novel approaches in molecular biology that employ CRISPR technology selectively remove specific proteins from the cell instead of inhibiting protein activity. This technology also has promising potential applications in therapy. In this case, the protein is targeted for degradation via the cell’s natural ubiquitin-proteasome pathway (UPS). Compounds such as molecular glues and proteolysis targeting chimeras (PROTACs) initiate this process by binding the target protein to an E3 ligase. Currently, there are many efforts to convert low potency inhibitors to PROTACS. These are small molecules consisting of two active domains and a linker capable of removing specific proteins from the cell (Figure 6.). PROTACs induce selective intracellular proteolysis and consist of two covalently linked molecules capable of binding proteins: One part binds an E3 ubiquitin ligase, and another part binds to the target protein to be degraded. Recruitment of the E3 ligase to the target protein results in ubiquitination and subsequent degradation of the target protein by the proteasome. PROTACs do not inhibit enzymatic activity but bind their target proteins with high selectivity [46,47].

PROTACs that can inhibit or degrade CDKs are also currently in development. Table 1 lists several PROTACs that have the potential to target and degrade cyclin-dependent kinases with minimal doses.

## 4. Development of Non-Selective Pan-CDK Inhibitors

Since cell cycle dysregulation may play a key role in tumor development, many different CDKIs have been developed as anti-cancer drugs. 

As the following section points out, the early developed CDKIs experienced only moderate clinical success with partly severe side effects. It was later concluded that the main reason for their failure was the functional diversity of individual CDKs. The use of non-specific CDK pan-inhibitors that addressed several CDKs simultaneously triggered uncontrollable effects which could ultimately cause more harm than good in cancer patients. 

In the following, the history of CDKI development, with all its successes, problems and limitations, will be discussed in more detail.

### 4.1. First Generation of CDK Inhibitors

**Figure 7:**
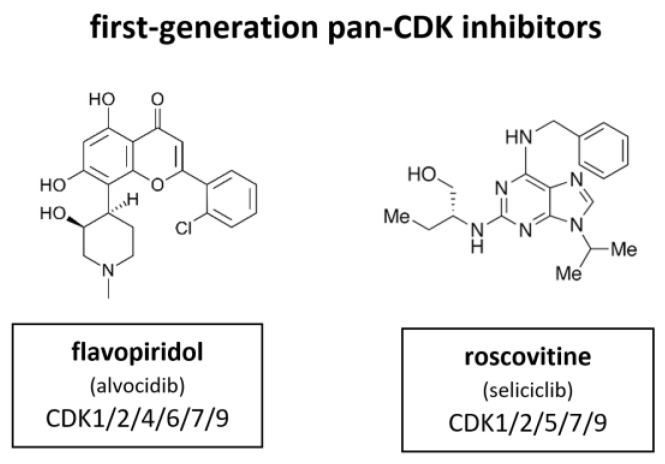
Structure of the first-generation pan-CDK inhibitors flavopiridol (also known as alvocidib) and roscovitine (also known as seliciclib).

First generation of CDKIs were non-specific pan-inhibitors (Figure 7). These agents induce G1 and G2 phase cell cycle arrest, finally leading to apoptosis. Initially, their effect was attributed to the inhibition of the cell cycle CDKs. However, later studies demonstrated that many of the cellular activities of these inhibitors were probably the result of CDK7 or CDK9 inhibition, the CDKs responsible for regulation of RNA transcription in the mitotic phase of cell division, as well as apoptosis-related genes (Figure 2) [61,62]. 

Flavopiridol, also known as alvocidib (Sanofi–Aventis), is the most extensively investigated CDKI among the first-generation inhibitors. Between 1998 and 2014 more than 60 clinical trials have been conducted. It has been shown to inhibit CDK1, CDK2, CDK4, CDK6, CDK7 and CDK9. In certain contexts it also induces a cytotoxic response, probably as a result of CDK7 and CDK9 inhibition that leads to suppression of RNA transcription [63,64,65,66]. Flavopiridol did not meet the high expectations for a CDKI in the clinical context. In contrary to the broad and substantial in vitro activity, there was quite little activity observed in in vivo studies [66]. Flavopiridol has demonstrated some clinical efficacy in hematological malignancies, such as chronic lymphocytic leukemia (CLL), but responses were limited by toxicity [67]. In Phase II studies flavopiridol did not meet necessary criteria although there is evidence that it may have clinical activity in hematological malignancies, such as CLL and mantle cell lymphoma (MCL) [68,69]. Scheduling seems to influence flavopiridol’s efficacy. Response rates as high as 41% in 22 assessable patients with CLL were mostly associated with short infusion time (4 h) [67]. Despite these reports and extensive investment, no Phase III studies have emerged and drug development of flavopiridol was consequently discontinued in 2012.

In parallel, a purine based CDKI called roscovitine, also known as seliciclib (Cyclacel), was evaluated in a Phase I trial with non-small cell lung cancer (NSCLC) patients. Roscovitine inhibits CDK1, CDK2, CDK5, CDK7 and CDK9. The plasma levels of roscovitine in phase I studies were not sufficiently sustained, as demonstrated in vitro [70]. Accordingly, attempts to measure Rb phosphorylation and cyclin D1 expression did not show reliable alteration of biomarkers under treatment [71]. The response rate was very low. Out of 56 only one single patient with hepatocellular carcinoma (HCC) achieved a partial response [72]. A subsequent blinded randomized phase II study (APPRAISE) with 187 patients was terminated. Its results were never published but the Cyclacel press release stated there was no difference between the seliciclib and placebo arms in terms of progression free survival (PFS) (48 versus 53 days respectively) but an increase in median overall survival was observed, favoring the seliciclib arm over the placebo arm (388 versus 218 days respectively). There was also a phase II, dose ranging, multicenter, randomized, double-blind, placebo-controlled study (ROSCO-CF) with 36 cystic fibrosis patients (24 treated, 12 controls). This study was terminated in 2018 [73]. Currently, a multicenter study of roscovitine is ongoing for Cushing’s disease (2021).

### 4.2. Second Generation of CDK Inhibitors 

The main drawback of the first-generation CDKIs flavopiridol and roscovitine was their lack of selectivity with concomitant adverse effects for patients while not having sufficient efficacy to prevent tumor progression. The focus of further development was therefore on increasing the selectivity of CDKIs. 

**Figure 8:**
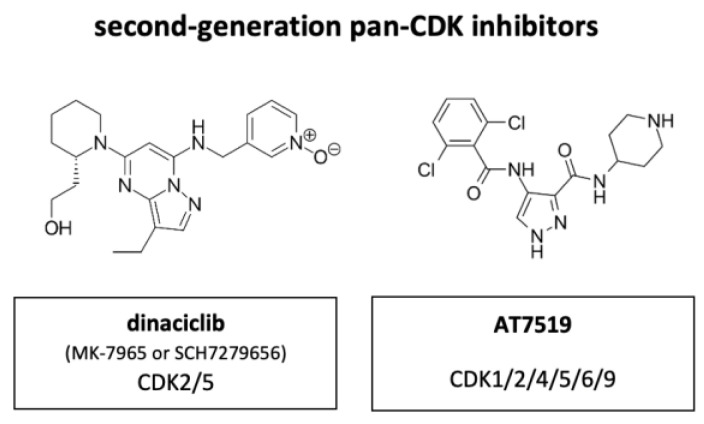
Structure of the second-generation pan-CDK inhibitors dinaciclib (also known as MK-7965 or SCH7279656) and AT7519.

Out of the next generation CDKIs dinaciclib, also known as MK-7965 or SCH7279656 (Merck, Darmstadt, Germany), has been most extensively studied clinically (Figure 8). Compared to flavopiridol, dinaciclib highly effectively targeted CDK2 and CDK5 (IC_50_ values of 1 nM each versus 12 and 14 nM with flavopiridol). IC_50_ of CDK1 and CDK9 were equally high between flavopiridol and dinaciclib with 3 nM and 4 nM each in ovarian carcinoma. The suppressive activity against CDK4, CDK6 and CDK7 was less, with IC_50_ values ranging between 60–100 nM. In vitro, dinaciclib completely suppressed Rb phosphorylation, which correlated with apoptosis onset and total inhibition DNA synthesis in >100 tumor cell lines of diverse origin and background. Furthermore, dinaciclib showed improved efficacy against solid tumors in a range of mouse models with doses below the maximally tolerated level [74]. Dinaciclib has shown some activity in MYCN-driven neuroblastoma, attributed to inhibition of CDK2 and CDK9. The overexpression of CDK2 in neuroblastoma tissue is associated with poor overall survival, suggesting a potential strategy for patient selection during clinical development of this drug [75].

An initial phase I trial on dinaciclib included 48 patients. Ten patients achieved a prolonged stable disease for at least four treatment cycles and it was classified as safe and well tolerated [76]. However, Phase II trials revealed disappointing results. 

A randomized Phase II trial with metastasizing breast cancer patients was terminated because the time to disease progression was inferior with dinaciclib treatment compared with capecitabine treatment. Moreover, grade 3 or 4 treatment-related adverse events were common and included neutropenia, leukopenia, increase in aspartate aminotransferase, and febrile neutropenia [77]. Additional randomized Phase II trials with NSCLC patients and patients with advanced acute myeloid leukemia (AML) (patients ≥60 years of age) or acute lymphoblastic leukemia (ALL) concluded that dinaciclib has no activity as monotherapy or did not show any objective responses with dinaciclib [78,79].

In contrast, a Phase II trial with patients with relapsed multiple myeloma (MM) demonstrated encouraging single-agent activities with two patients achieving a deep response and ten patients obtaining some degree of M-protein stabilization or decrease [80]. Furthermore, dinaciclib proved useful in the treatment of specific hematological malignancies. 

A Phase III study, which was terminated in 2017, concluded that dinaciclib demonstrated an acceptable and tolerable safety profile compared to ofatumumab, a human B-cell inhibitory monoclonal antibody targeting CD20, in patients with relapsed/refractory CLL. In contrast to first-generation CDKIs, tolerability of dinaciclib, appeared to be greatly improved [81]. 

More clinical trials on CDKIs include AT7519 (Astex), an inhibitor of CDK1, CDK2, CDK4, CDK5, CDK6 and CDK9. Treatment with AT7519 induces cell cycle arrest in G0/G1- and G2/M-phase. The compound has excellent anti-tumor efficacy in human colorectal cancer xenograft models, with extensive tumor regression, impact on pharmacodynamic biomarkers and increased PARP cleavage, indicative of apoptotic cell death [82].

Several Phase I/II trials were conducted in patients with previously treated MM, with relapsed MCL, CLL, advanced or metastatic solid tumors or refractory non-Hodgkin’s lymphoma (NHL). None of these studies progressed beyond phase II. Only indirubin entered Phase IV stage. Table 2 lists most of the pan-CDKIs that have entered a clinical trial (2021). Some of the listed compounds like AG024322 were not proven to be clinically efficacious [83].

Indirubins are bisindole alkaloids that naturally occur in indigo-containing plants such as *Indigofera tinctoria* L. and *Isatis tinctoria* L. as well as in mollusks of the family *Muricidae*. They belong to the rather small family of indigoids, which, however, has become very important in the medical field. Indirubin has been found to be the active ingredient of a traditional Chinese medicine. In the 1980s, indirubins were clinically tested for the treatment of chronic myeloid leukemia (CML). More than 50% of CML patients experienced partial or complete remission [84], similar to standard treatment with the cytostatic drug busulfan [85]. Furthermore, indirubin toxicity was low and side effects, which occurred in about half of the participants. These encouraging results encouraged researchers to investigate the use of indirubin and its novel derivatives in other cancers and diseases. 

Indirubin-3-oxime can inhibit the kinase activity of CDK9 to block tyrosine aminotransferase-mediated expression of human immunodeficiency virus RNA, thereby inhibiting replication of wild-type and drug-resistant HIV-1 [86]. Indirubin-3’-monoxime derivatives serve as potent inhibitors of CDK2 and CDK9 [87]. Indirubin derivate E211, which has already successfully been tested in the clinic, exhibits relatively low inhibitory activity towards various CDKs. Introduction of a sulphonate group into position 5 (E226) very strongly increases inhibitory activity with an IC50 value toward CDK1/cyclinB of only 5 nM [88]. Unfortunately, E226 was found not to penetrate cellular membranes to any measurable extent and therefore did not show comparable inhibitory effects towards CDK in cells, nor did it show any cell growth inhibition. When the sulphonate group was replaced by a N,N-dimethyl sulphonamide group (E233) the inhibitory potency towards isolated CDK1 and CDK2 remained extremely high. Still, the compound was found not to be taken up sufficiently into tumor cells, and therefore showed only poor growth inhibition [89]. Indirubin is also known to be a potent inhibitor of CDK6, the Ser/Thr kinase with regular activity in the cell cycle. It exhibits high binding affinity [90]. In total, there are six clinical studies on indirubin for patients with psoriasis vulgaris, nail psoriasis, atopic dermatitis and Childhood Acute Promyelocytic Leukemia. For acute promyelocytic leukemia (APL) there is an ongoing phase 4 clinical trial (NCT02200978). These studies showed that this agent was effective against APL. Indirubin is relatively inexpensive and can be taken orally, resulting in reducing the number of hospital days and the treatment cost.

### 4.3. Problems and Limitations of Pan-CDK Inhibition

There are several reasons that may explain why the non-selective inhibitors did not lead to success in clinical application. The lack of specificity is the most obvious one. Especially the consequential insufficient understanding of the complex underlying mechanisms of action of the respective drugs makes the failure hard to explain. This lack of understanding hinders the progression of these agents to be targeted therapies and the development of effective combination strategies. Moreover, due to their non-specific mode of action, a wide variety of cellular effects occur. This fact makes it difficult to predict the final therapeutic outcome in the patient. It is indeed these multiple and diverse, unpredictable mechanisms of action that lead to normal cells being affected in addition to tumor cells. This in turn creates a strong impediment to achieving a proper therapeutic window and therapeutically effective concentrations high enough to destroy tumor cells. Accordingly, there have been side effects and toxicities associated with non-selective CDKIs including diarrhea, myelosuppression, anemia, hepatic dysfunction and nausea [72,76,91].

Another important reason for the missing clinical success of non-specific CDKIs is the lack of biomarkers to select patient subpopulations which were particularly sensitive to CDKIs. Clinical trials showed that there were some exceptional responders, but it was inconclusive what the exact underlying condition was. It is possible—we are seeing this in our research—that the mechanism of action is highly dependent on the hetero- or homogeneity of the tumor mass as well as the molecular cell cycle machinery, which varies depending on the tumor environment. So, it could be assumed that there are certain time frames which are important for therapeutic use.

Although non-specific pan-CDKIs have shown disappointing clinical results in a broad range of applications, there is nevertheless some evidence that non-specific pan-CDKIs are also actually effective in a limited range of applications. The known limitations of pan-CDKIs suggest that improving selectivity for specific CDKs is pivotal to the successful development of CDKIs as cancer therapeutics. With the development of specific CDKIs, the potential for these drugs to be deployed in broad clinical use gained great momentum.

Interestingly, recent studies have shown that combination therapies, in contrast to CDKI monotherapies, can provide promising results in tumor therapy. In some tumor entities, a combination of conventional treatment and CDK inhibition has already been shown to reverse treatment resistance, such as radio- or chemoresistance. This relationship will be discussed in more detail later along in this review. It is particularly discussed for HNSCC, a tumor entity with little improvement in treatment outcomes for decades despite medical advances in recent years. 

## 5. Development of Selective CDK Inhibitors 

Due to their ubiquitous biological functions pan-selective CDKs are of limited use as clinical targets. Complex pharmacokinetics and dose-limiting toxicities have complicated the use of CDKIs. Due to the insurmountable adverse effects and reduced activity observed in vivo, the development of many CDKIs, which initially displayed reliable results in vitro and in Phase I studies, was eventually discontinued. Because of the narrow therapeutic window, specific CDKIs are necessary to provide an effective cancer treatment with reduced side effects. Some of them have already been evaluated for their efficacy, experimentally as well as clinically. For an overview of selected specific CDKIs see Table 3. Some of the specific CDKIs are currently investigated in ongoing clinical trials, as indicated in the table. 

### 5.1. CDK1 Inhibitors

**Figure 9:**
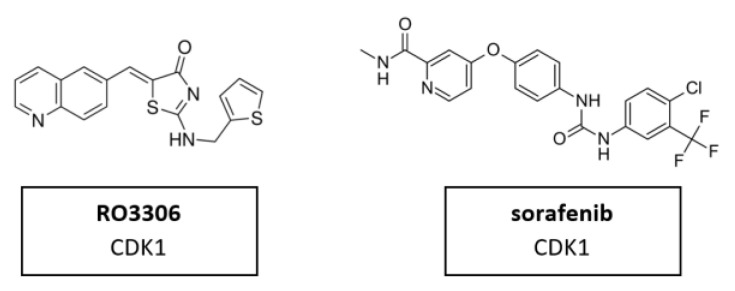
Structure of CDK1 inhibitors RO3306 and sorafenib.

CDK1 is one of the main cell cycle regulators and a key factor of mitosis. A CDK1 knockdown is already lethal in the early cell division stages [115]. Therefore, CDK1 inhibition may be toxic in certain contexts. It may also be difficult to find a therapeutic window for CDK1 inhibitors in which exclusively tumor cells are targeted, excluding the normal, non-malignant cells. Targeting CDK1 might be selectively lethal to MYC-dependent human breast cancer cells [116]. Nevertheless, a limited number of studies have been conducted. CDK1 inhibitor RO3306 in combination with sorafenib treatment significantly decreased tumor growth in patient derived xenograft (PDX) tumor models. Furthermore, the combinatorial treatment could overcome sorafenib resistance in a preclinical PDX model of HCC [93]. The molecular structure of CDK1 inhibitors RO3306 and sorafenib are shown in Figure 9.

### 5.2. CDK2 Inhibitors

**Figure 10:**
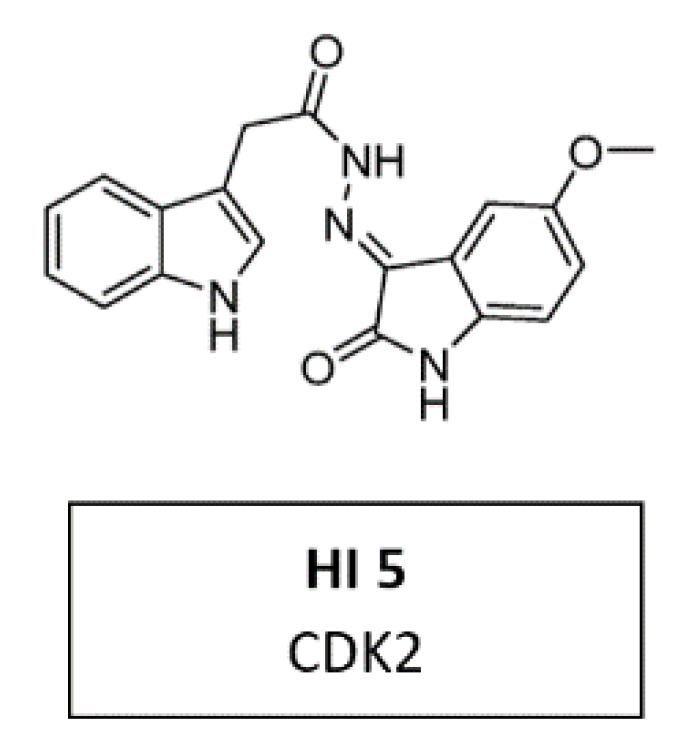
Structure of CDK2 inhibitor HI 5.

Due to its crystallographic structure availability, initial efforts for second-generation CDKIs were primarily focused on inhibition of CDK2, which is essential for DNA replication [94]. A recent in vitro study demonstrated a new CDK2 inhibitor with 3-hydrazonoindolin-2-one scaffold, called HI 5 (Figure 10). This inhibitor induced intrinsic apoptosis in human breast cancer cell line MCF-2 [117].

Since CDK7, CDK8/19, CDK12/13, or CDK9 are associated with basal RNA transcription, it seemed a reasonable strategy to selectively target these CDKs, as cancer cells may be particularly susceptible to selective suppression (Figure 8). 

The following CDK inhibitors affect CDKs which are thought to be relevant in regulating the action of RNA polymerase II (Figure 3).

### 5.3. CDK4/6 Inhibitors

**Figure 11:**
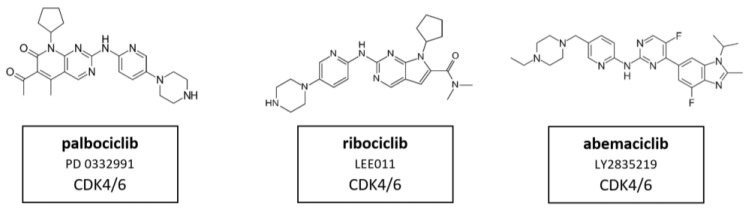
Structure of the specific CDK4/6 inhibitors palbociclib (also known as PD 0332991), ribociclib (also known as LEE011) and abemaciclib (also known as LY2835219).

The development of selective inhibitors, of both CDK4 and CDK6, has markedly changed the perception of CDKs as therapeutic targets in cancer. Dual CDK4 and CDK6 inhibitors have been shown to be active in multiple preclinical models, including xenografts, genetically engineered mouse models and primary human tumor explants. Parallel drug discovery efforts at Pfizer, Eli Lilly and Novartis resulted in the development of the drugs palbociclib (PD-0332991), ribociclib (LEE011) and abemaciclib (LY2835219), respectively (15) [118,119]. Each drug is structurally similar, and the selectivity of these compounds is characterized by binding to the specialized ATP-binding pocket of CDK4 and CDK6 and specific interactions with residues in the ATP-binding cleft [120]. All three compounds were evaluated through chemical screening and optimization by adding pyrido[2,3-d] pyrimidin-7-one compounds with a side chain of 2-amino pyridine at the C2 position of CDKIs. The pyrido[2,3-d]pyrimidin-7-one template has been identified previously as a privileged structure for the inhibition of ATP-dependent kinases [121]. Palbociclib, ribociclib, and abemaciclib are administered orally and have limited suppression of other CDK activities at clinically achievable doses. These inhibitors are structural analogues to flavopiridol, but have different chemical functions [122]. CDK4/6 inhibitors contribute to G0/G1 phase cell cycle arrest by preventing the phosphorylation of pRb which leads to the inhibition of tumor progression in a variety of cancers (see Figure 1 and Figure 2). Therefore, it is assumed that in HNSCC, CDK4/6 inhibitors showed best results in HPV-negative HNSCC with high Rb expression and an epithelial phenotype. Despite oncogenic signals that are insensitive to endocrine therapy, CDK4/6 inhibitors effectively prevent cell cycle progression and mitosis [123]. These three CDK4/6 inhibitors are described in more detail in the following paragraphs.Their molecular structure is shown in Figure 11.

#### 5.3.1. CDK4/6 Inhibitor Palbociclib

Palbociclib, also known as PD 0332991, was the first CDK4/6 inhibitor to be approved in cancer therapy [124]. This selective CDK4/6 inhibitor prevents phosphorylation of the Rb protein and subsequently downregulates the E2F-driven gene, leading to cell cycle arrest in G1 phase. Additionally, it downregulates Ki-67 expression in Rb-positive models. In xenografts of Rb-negative tumors this effect could not be detected [125]. Interestingly, it was found to sensitize cancer cells to other therapeutic treatments when applied as combination therapy, such as chemotherapy [123] and ionizing radiation [126]. The potent anti-tumor effects of palbociclib have been observed in several tumor types, including T cell acute lymphoblastic leukemia (T-ALL) [127], HCC [128], neuroblastoma [129], RCC [130], myeloma [131], MCL [132], pancreatic ductal adenocarcinoma [133], esophageal adenocarcinoma [134], medulloblastoma [126], melanoma [135], NSCLC [136], and particularly in breast cancer [137]. Palbociclib was approved by the FDA in 2015 in combination with letrozole as initial hormone- plus CDK-targeted therapy for post-menopausal women with ER(+)/Her2(−) advanced breast cancer [95]. Moreover, the therapeutic effects of palbociclib have been demonstrated in numerous clinical trials for breast cancer, NSCLC, Goodpasture’s Syndrome, lymphoma and leukemia, in combination with 5-FU and oxaliplatin in solid malignancies (NCT01522989) or with cetuximab, a monoclonal antibody against the epidermal growth factor receptor (EGFR), in HNSCC [138,139].

Palbociclib has also been studied in multiple animal models of a wide variety of tumor types, including liver cancer [128], glioblastoma multiforme [140], pancreatic neuroendocrine tumors [141], gliomas [142], colon carcinoma [143] breast cancer [144]. This compound led to growth arrest of xenograft tumors and prolonged survival of treated animals. Mice models with ERBB2-overexpressing breast carcinoma showed that CDK4/6 was pivotal to the maintenance of the disease.

Phase I trials evaluated palbociclib in patients with Rb-positive tumors. These studies exhibited the dose-limiting toxicity (DLT) of palbociclib to be neutropenia and the maximum tolerated dose (MTD) was 125 mg daily for 21 days per 28-day cycle or 200 mg daily for 14 days per 21-day cycle [145,146].

Currently there is an ongoing biomarker-based study called UPSTREAM, of the European Organization for Research and Treatment of Cancer—EORTC, for HNSCC. The umbrella biomarker-driven study is dedicated to recurrent and/or metastatic HNSCC patients. It is the first multicenter pilot study proposing a therapeutic strategy based on biomarkers in patients with recurrent/metastatic HNSCC, palbociclib is indicated in patients with cyclin D1 (CCND1) amplification and p16 negativity [147].

#### 5.3.2. CDK4/6 Inhibitor Ribociclib

The mode of action of ribociclib, also known as LEE011, has been investigated in numerous in vitro studies, particularly in cancers such as leukemia [148], neuroblastoma [149], neuroendocrine tumors [150], liposarcomas [151], breast cancer [152] and HNSCC [153].

The efficacy of ribociclib was also assessed in animal models bearing multiple cancer types including breast cancer [154], neuroblastoma [129], liposarcoma [151] and HNSCC. Tumor size stabilization was observed in three HPV-negative PDTX models and tumor growth was delayed in one HPV-negative tumor compared with controls. Although its volume increased over time under ribociclib treatment two tumors were resistant to the CDKI treatment [153].

#### 5.3.3. CDK4/6 Inhibitor Abemaciclib

Abemaciclib, also known as LY2835219, is the third selective CDK4/6 inhibitor, which has been studied in several tumors to date, including breast cancer [155], melanoma [156], bladder cancer [157] and oral squamous cell carcinoma (OSCC). In OSCC, this inhibitor was evaluated in a phase I study. The clinically most significant adverse event was grade 3 fatigue. The maximum tolerated dose was 200 mg every 12 h. Current data suggest that abemaciclib can penetrate the central nervous system with potential interest in the treatment of brain metastases. At the molecular level, abemaciclib inhibits the activation of AKT and ERK, but not mTOR. Abemaciclib has also been reported to decrease ABC transporter-induced resistance of tumor cells to chemotherapeutic agents. Its treatment significantly sensitized ABCB1 or ABCG2 overexpressing cancer cells by impairing the functions of ABCB1 and ABCG2 in these transporter-amplified cancer cells [155]. The combination of abemaciclib with everolimus, an mTOR inhibitor, proved to have more efficacy than abemaciclib in monotherapy in a recent study and seems a promising therapeutic approach for HNSCC [158].

CDK4/6 inhibitors may also exert systemic effects and can therefore lead to adverse side effects such as thrombosis or pulmonary embolism. In addition, the formation of resistance has been reported [159,160]. These limitations led to the exploration of alternative strategies to reduce increased kinase activity in cancer cells.

#### 5.3.4. CDK4/6 Inhibitor Trilaciclib

**Figure 12:**
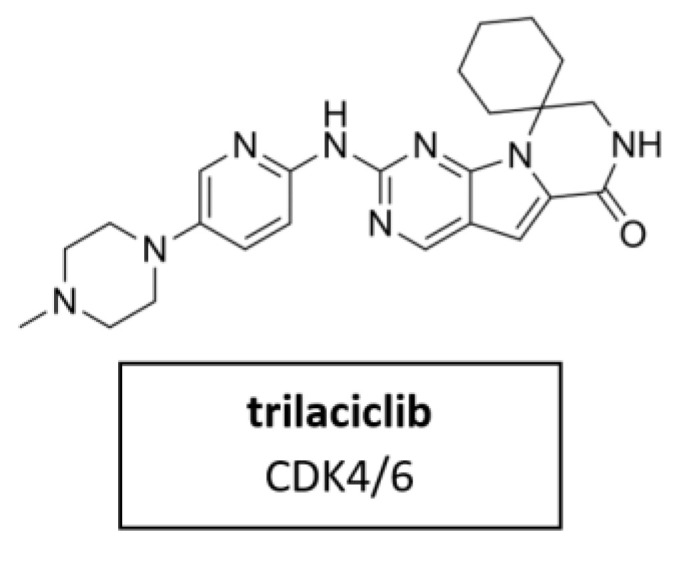
Structure of the novel specific CDK4/6 inhibitor trilaciclib.

Trilaciclib (Figure 12) is a small molecule, short-acting CDK4/6 inhibitor. It was proven for its myeloprotection and potential antitumor efficacy and safety in combination with cancer chemotherapy [98]. In February 2021, trilaciclib received its first approval in the U.S. to reduce chemotherapy-induced myelosuppression in adult patients when administered prior to platinum/etoposide-containing or topotecan-containing chemotherapy for advanced-stage small cell lung cancer (ES-SCLC) [161]. Clinical trials in breast cancer, colorectal cancer and small cell lung cancer are currently underway in several countries.

### 5.4. CDK7 Inhibitors

**Figure 13:**
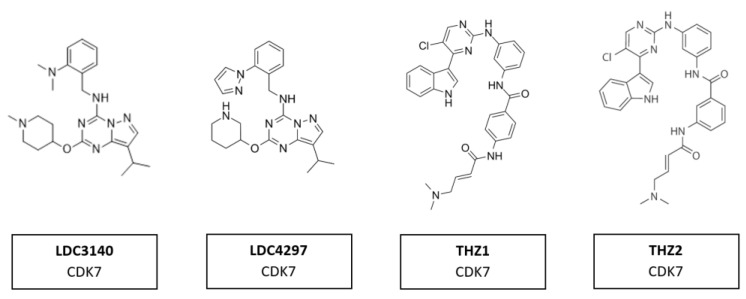
Structure of CDK7 inhibitors LDC3140, LDC4297, THZ1 and THZ2.

Several compounds have shown potential as promising selective inhibitors of CDK7. These include LDC3140 and LDC4297, THZ1 and THZ2 (Figure 13). The high specificity of these inhibitors derives from their ability to interact with a cysteine residue outside the catalytic domain of CDK7, which is absent in other CDKs. These inhibitors, when used to treat tumor cells, showed preferential impacts on RNA polymerase II activities [103]. They have anti-tumor activity in multiple tumor types, including aggressive and heterogeneous cancers, such as neuroblastoma, small cell lung cancer and triple-negative breast cancer, with poorly defined oncogenic driver mutations [162]. These tumors had in common an amplification of one of the MYC family members. Regions of high transcription activity have clusters of enhancers, known as super-enhancers. It is assumed the responsiveness of cells to these inhibitors are due to the sensitivity of super-enhancers to CDK7 inhibition [103].

#### CDK7 Inhibitor SY-5609

**Figure 14:**
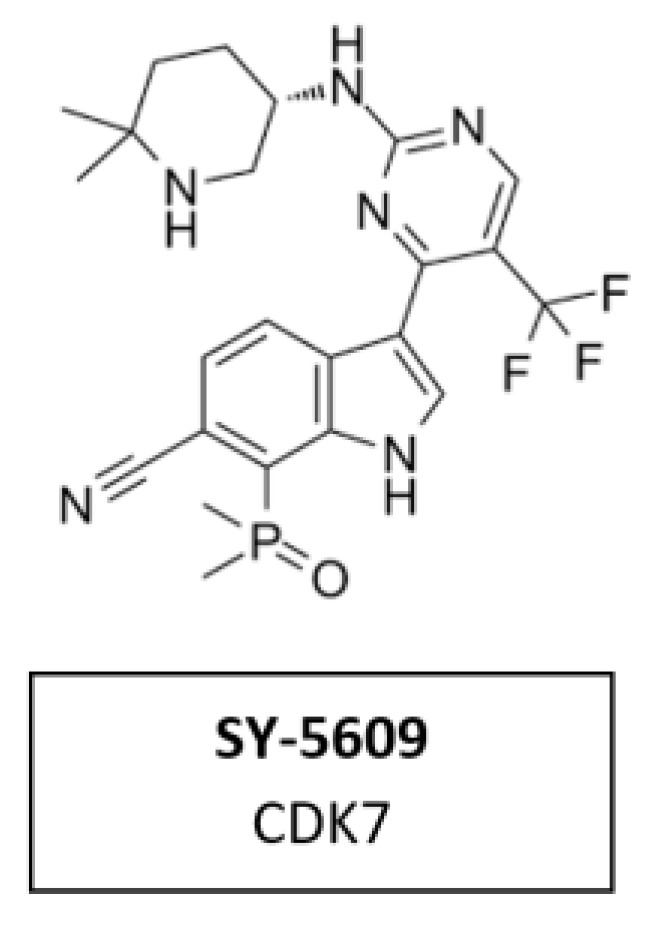
Structure of the specific CDK7 inhibitor SY-5609.

CDK7 has emerged as a target of interest in oncology due to its role in two important processes that are dysregulated in cancer cells—cell cycle and transcription. SY-5609 (Figure 14) the discovery of a highly potent (sub-nM) and selective orally available CDK7 inhibitor. It was introduced to the clinic in 2020 (NCT04247126). Structure-based design was leveraged to obtain high selectivity (>4000-times the closest off target) and slow off-rate binding kinetics desirable for potent cellular activity. The incorporation of a phosphine oxide as an atypical hydrogen bond acceptor helped to achieve the required potency and metabolic stability. SY-5609 exhibits potent inhibition of CDK7 in cells and demonstrates strong efficacy in mouse xenograft models at doses as low as 2 mg/kg [102].

### 5.5. CDK8/19 Inhibitors

**Figure 15:**
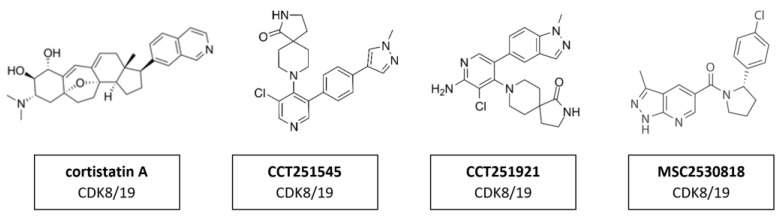
Structure of CDK8/19 inhibitors cortistatin A, CCT251545, CCT251921 and MSC2530818.

The transcription regulators CDK8 and CDK19 have 91% sequence homology over the first 370 residues—which encompass the active site—but differ substantially toward the C-terminus in areas that may contribute to their non-redundant functions [163]. This suggests that the active site of those two proteins is remarkably similar and compound selectivity is a challenge. A whole series of CDK8/CDK19 inhibitors have been found. Cortistatin A (Figure 15) is a steroidal alkaloid isolated from the marine sponge *Corticium simplex*. Cortistatin A has a 17 nM binding affinity to CDK8 and 10 nM to CDK19 [104]. A further study identified anti-leukemic activity of cortistatin A, in vitro and in vivo, which inhibited CDK8/19 and induced the expression of superenhancer-associated genes in sensitive cell lines [105]. Additional studies revealed CCT251545 (Figure 15), a type I binding molecule, as potent small-molecule inhibitors of WNT signaling with a single-digit IC_50_ for CDK8 of 5 nM and CDK19 of 6 nM [106]. Additional CDK8/19 inhibitors have been reported in the literature, including a series of 6-aza-benzothiophene containing compounds that were developed into potent selective Type I inhibitors of CDK8 [107]. After the discovery of CCT251545 follow-up work yielded a 3,4,5-trisubstituted-2-aminopyridine series exemplified by CCT251921 (Figure 15). This compound is a potent selective and orally bioavailable inhibitor of CDK8, with equal affinity for CDK19 and optimal biochemical, pharmacokinetic and physicochemical properties [108]. However, the latter exhibited a pleiotropic toxicological profile, which rendered it impossible to find a therapeutically useful window in clinical trials. Further studies led to the discovery of MSC2530818 (Figure 15) a compound with excellent kinase selectivity, biochemical and cellular potency, microsomal stability and oral bioavailability [109]. However, the latter exhibited a pleiotropic toxicological profile, which rendered it impossible to find a therapeutically useful window in clinical trials. 

### 5.6. CDK9 Inhibitors

**Figure 16:**
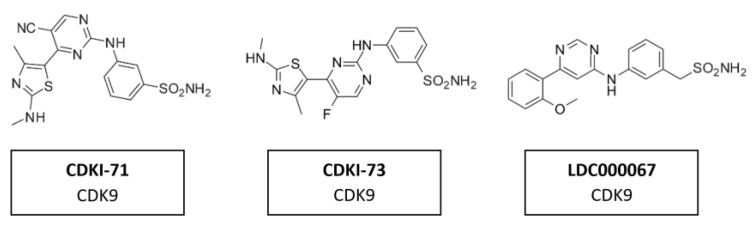
Structure of CDK9 inhibitors CDKI-71, CDKI-73 and LDC000067.

A range of CDK7/9 inhibitors with different selectivity profiles have been identified. This included CDKI-71 and CDKI-73 (Figure 16), a novel class of 5-substituted-4-(thiazol-5-yl)-2-(phenylamino) pyrimidines [164]. CDKI-71 showed cytotoxicity and induced caspase-dependent apoptosis in human cancer cell lines, primary patient leukemia cells, B and T cells as well as embryonic lung fibroblasts. Notably, compared with the unselective alvocidib, CDKI-71 preferentially affected cancer cells [165]. Treating primary human leukemia cells with CDKI-73 has been shown to lead to the dephosphorylation of CDK9, the dephosphorylation of the RNA polymerase II CTD at Ser2 and to induce caspase-dependent apoptosis. CDKI-73 was more potent than the pan-CDKI alvocidib, showing selectivity for primary leukemia cells over normal CD34+ cells and was synergistic with fludarabine [166]. Treatment with LDC000067 (Figure 15) has been shown to increase the pausing of RNA polymerase II and to lead to a selective reduction in short-lived mRNAs, including those encoding regulators of proliferation and apoptosis. Nemeth et al. evaluated a highly potent inhibitor which was proven to be highly specific to CDK9/CycT1 and acts in an ATP-competitive manner. The inhibitory capacity of the compound and its high specificity for CKD9/CycT1 is exceptional. Results suggested it would have the potential to control HIV-1 replication in vivo while having a lower risk of inhibiting other kinases and consequently causing undesired toxicity [167].

### 5.7. CDK9 Inhibitor AZD4573

**Figure 17:**
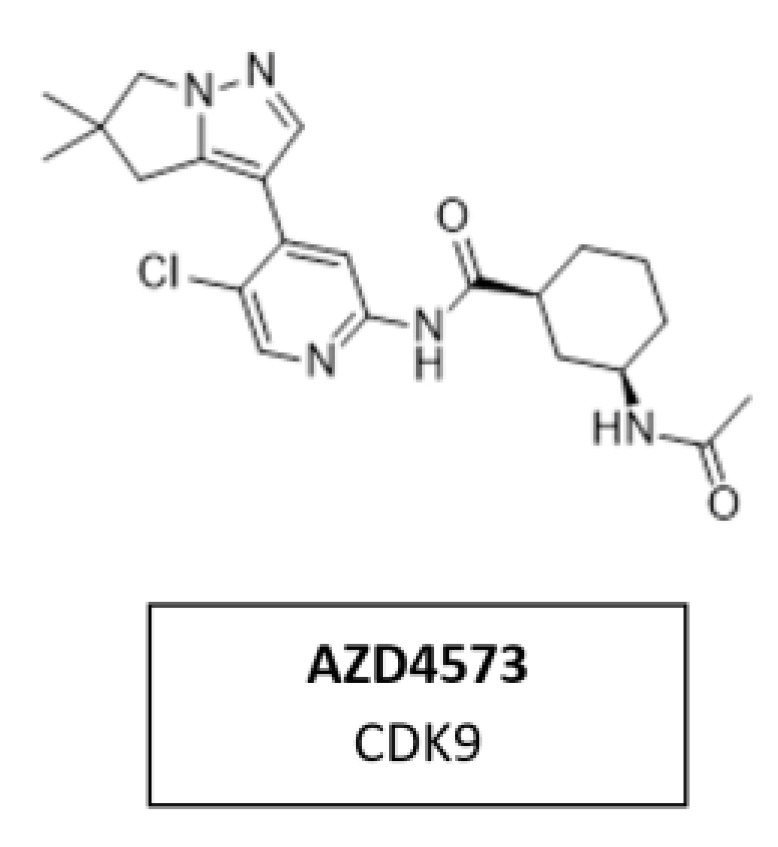
Structure of the specific CDK9 inhibitor AZD4573.

CDK9 is a transcriptional regulator and potential therapeutic target for many cancers. Multiple nonselective CDK9 inhibitors have progressed clinically but were limited by a narrow therapeutic window. AZD4573 (Figure 17) is a novel, potent, and highly selective CDK9 inhibitor. It enables the indirect inhibition of MCL-1, providing a therapeutic option for MCL-1-dependent diseases. Currently, AZD4573 is being evaluated in phase I and II clinical trial for patients with hematologic malignancies (NCT03263637, NCT04630756, NCT05140382) [168].

#### CDK12/13 Inhibitors

**Figure 18:**
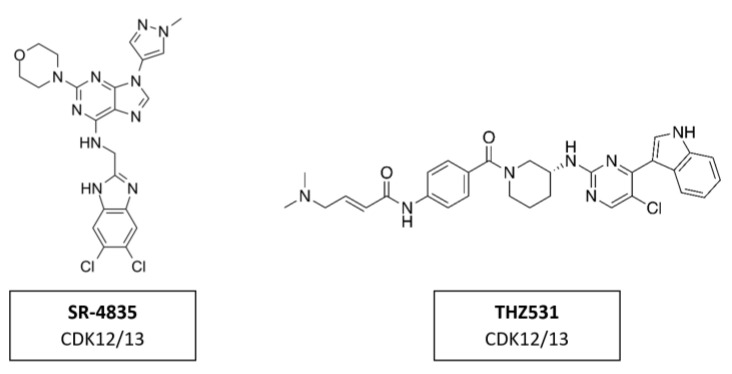
Structure of CDK12/13 inhibitors SR-4835 and THZ2531.

CDK12 phosphorylates the CTD region of RNA polymerase II promoting transcription elongation [169]. Several inhibitors, like SR-4835 and THZ531 (Figure 18), demonstrated strong anti-tumor activity in preclinical studies. SR-4835 is a highly selective dual inhibitor of CDK12 and CDK13, which can suppress the expression of core DNA damage response proteins and can thereby promote a synergistic effect of DNA damage chemotherapy and PARP inhibitors in triple negative breast cancer [170]. THZ531 is another covalent inhibitor of CDK12 and CDK13, which can significantly down-regulate the expression of DNA damage response genes and key super-enhancer-related transcription factors [114]. THZ531 has a synergistic effect with sorafenib in the treatment of hepatocellular carcinoma [171]. To date, the inhibitors targeting CDK12 in clinical trials have all been pan-CDKIs, including dinaciclib.

### 5.8. Problems and Limitations of Selective CDK4/6 Inhibitors

#### Resistance Mechanisms

Primary drug resistance is a clinical situation in which a malignant tumor does not respond to chemotherapy. Approximately 10% of patients will have primary resistance to CDK4/6 inhibitors. In the future, biomarkers may have the potential to identify these types of patients at baseline or shortly after treatment initiation, facilitating earlier switch to a more effective treatment. For instance, patients with evidence of functional Rb loss at baseline are not likely to benefit from CDK4/6 inhibition. For breast cancer, various preclinical studies suggested loss of Rb to be a driver of resistance to CDK4/6 inhibitors [172,173]. Similarly, baseline evidence of increased cyclin E1 expression or the CCNE1/RB ratio may also play a role in identifying these patients [159,174].

Peripheral evidence of ongoing neoplastic proliferation, as manifested by a rise in TK1 activity within a month of commencing therapy, may also provide a marker of early resistance.

One question that remains to be explored concerns the ongoing treatment with CDK4/6 inhibitors after disease progression with these compounds. The prospect that continuing a CDK4/6 inhibitor beyond disease progression may be an effective strategy is being explored in several ongoing phase 1 and phase 2 trials (MAINTAIN NCT02632045, PACE NCT03147287, NCT01857193, NCT 02871791, and TRINITI-1 NCT 02732119). Mutations in *RB1*, resulting in activation of other cell cycle factors, such as E2F and the Cyclin E/CDK2 axis, has been demonstrated in cases of acquired CDKIs resistance, which by definition would qualify as a secondary resistance. [175,176]. This in turn results in independence from the CDK4/6 pathway for cell cycle progression from G1 to S phase. In such cases, in the setting of disease progression on a CDK4/6 inhibitor, concurrent biomarker evidence of a functional loss of Rb may support a switch to a new agent, rather than continuing CDK4/6 agents beyond progression. Moreover, the elevation of CDK6 has also been associated with resistance to CDK4/6 inhibitors, revealing an additional potential mechanism of resistance [176,177]. Furthermore, the PI3K/mTOR pathway has been shown to be upregulated in response to chronic exposure to CDK4/6 inhibitors, which in turn upregulates cyclin D. In the absence of CDK4 and CDK6, activated cyclin D can activate CDK2, which subsequently drives cell cycle progression [159]. Functioning downstream of PI3K is 3-phosphoinositide-dependent protein kinase 1 (PDK1), a vital requisite for the full activation of AKT [178]. The PI3K-PDK1 signaling pathway has been implicated in mediating resistance to CDK4/6 inhibitors, with ribociclib-resistant breast cancer cell lines demonstrating an increase in PDK1 levels following drug exposure, resulting in activation of the AKT pathway [179].

## 6. Synergistic Effects of CDKI Treatment with a Focus on HNSCC

### 6.1. General Description of Molecular Settings Their Response to CDK Inhibitors

In the following chapter, the advantages of combining the partly quite disappointing outcomes of CDKI monotherapy together with standard therapies such as radio-, chemo-, targeted or even immunotherapy in various tumor types will be highlighted. Indeed, there is a clear tendency towards a synergistic effect of CDKIs, which may ultimately lead to sensitization of radio- and chemoresistance as well as a sensitization to immune checkpoint blockade. The synergistic effects that contribute overcoming resistance have been observed in several experimental studies and early clinical trials.

The synergistic effect of CDKIs in combination with radio-, chemo-, targeted or even immunotherapy effect has been reported in HNSCC, as well as in ovarian, prostate, and non-small cell lung cancer. In the following, we particularly focus on HNSCC. HNSCC represents the eighth most common form of cancer worldwide. Nearly 800,000 new diagnoses are made annually, with a large increase in incidence over the past 10 years. Despite remarkable progress over the last decades, the 5 year overall survival rate of patients with HNSCC is still about 50% [3]. For targeted treatment, HNSCC need to be divided into two major subgroups with respect to their oncogenesis, resulting in molecular differences: human papillomavirus (HPV)-negative and HPV-positive HNSCC.

In HPV-negative HNSCC, the CCND1/CDK4/6-CDKN2A (p16^INK4A^) Rb pathway is affected by amplification of CCND1 (in 20–30% of cases) and inactivation of p16^INK4A^ (in approximately 90% of cases), which results in inactivation of the pRb protein by hyperactivation of the cyclin D/CDK4/6 complex [180]. CDKN2A, that encodes cyclin-dependent kinase inhibitor p16^INK4A^, is frequently inactivated by a copy number loss in HPV-negative HNSCC [181,182]. In contrast, p16^INK4A^ overexpression is typical for HPV-positive HNSCC [17] and used as a surrogate marker of HPV infection [182].

It has been shown that CCND1 amplification and inactivation of p16^INK4A^ are markers for poor prognosis in HNSCC partly because overexpression of CCND1 decreases tumor response to cisplatin [183] and EGFR inhibitors [184].

In HPV-positive HNSCC, the viral oncogenes E6 and E7 are associated with reduced pRb and p16^INK4a^ overexpression. Wiest et al. showed that the E7 oncogene of the HPV16 subtype induces a functional inactivation of pRb [185]. Consequently, CDK4/6 inhibitors may only be beneficial in patients with p16/HPV16-negative HNSCC.

The finding that the CCND1/CDK4/6-CDKN2A (p16^INK4A^) Rb axis is altered in a substantial number of HNSCC cases provides hope for specific CDK4/6 inhibitors to achieve good therapeutic efficacy.

### 6.2. Clinical Phase Trials with CDKI Combination Therapies

This discovery of specific CDK targets will hopefully expand treatment options for patients by using CDKIs in monotherapy or in combination with radio-, chemo-, and immunotherapy, preventing treatment resistance. The addition of CDK4/6 inhibitors to several established treatments for HNSCC has the potential to improve responses to other therapies and may help overcome resistance.

CDK4/6 inhibitors in monotherapy have demonstrated cytostatic activity in HPV-negative HNSCC. However, several studies on monotherapies have shown potent anti-tumor activity and manageable toxicity in HR+/HER2−breast cancer patients. In contrast monotherapy in HNSCC demonstrated only relatively poor results so far [97]. Despite preclinical success, monotherapies targeting EGFR or cyclin D1-CDK4/6 in HNSCC have shown a limited clinical outcome [186].

At present, several clinical trials of CDK4/6 inhibitors are ongoing for patients with HNSCC. Table 4 lists the most important clinical trials currently in progress.

### 6.3. Influence of CDKIs on Radiosensitivity

#### 6.3.1. CDK4/6 Inhibitor Flavopiridol Combined with Radiotherapy

Flavopirodol is the most extensively studied CDKI among first-generation nonspecific CDKIs. However, it never reached phase III trials and was therefore discontinued in 2012. Despite its rather disappointing success in monotherapy, a surprising sensitization to radiotherapy has been observed.

Data from the literature show that CDKIs from all stages of pharmaceutical development, alter or increase the sensitivity of various tumor types to radiation. Raju et al. demonstrated an in vitro radiosensitizing effect of flavopiridol (300 nM for 1 day) and the underlying molecular mechanisms in a murine ovarian cancer cell line, OCA-I. After flavopiridol treatment clonogenic assays showed in vitro inhibition of repair from radiation damage after split-dose radiation.

Mechanisms behind that may comprise influencing the expression levels of proteins involved in DNA repair processes and accumulation of cells in G1 and G2 phases with significant reduction of S phase. Among cyclin D1 and cyclin E, flavopiridol downregulated CDK9, the catalytic subunit of positive transcription elongation factor b (P-TEFb), suggesting that flavopiridol may modulate cellular transcription processes [187].

This modulation of transcription by flavopiridol with a simultaneous radiosensitization effect by reduction in phosphorylation of RNA polymerase II was demonstrated by the same group in human esophageal adenocarcinoma cells and xenograft experiments. Flavopiridol, either given before or after radiation in xenograft experiments with nude mice greatly enhanced the effect of tumor irradiation [188].

Additionally, flavopiridol enhanced radiosensitivity of prostate cancer cell lines. Flavopiridol modified the time course of gammaH2AX expression, a sensitive molecular marker of DNA damage and repair, in irradiated cells. The number of cells expressing gammaH2AX foci was significantly greater in flavopiridol-treated cells. These results indicate that flavopiridol can enhance radiosensitivity of human tumor cells and suggest that this effect may involve an inhibition of DNA repair [189].

#### 6.3.2. CDK4/6 Inhibitor Palbociclib Combined with Radiotherapy

Palbociclib was the first specific CDK4/6 inhibitor to be approved in cancer therapy [124]. A recent study in HNSCC by Göttgens et al. demonstrated enhanced radiosensitivity after inhibition of CDK4/6. The clinically approved inhibitor palbociclib exerted radiosensitization effect via decrease of BRCA1 and RAD51 induction, regulators of DNA damage repair, after irradiation. In the presence of palbociclib homologous recombination was diminished and repair of radiation-induced DNA damage was delayed leading to increased chromosomal damage [190]. Furthermore, a combined pharmacological inhibition of MEK and CDK4/6 led to substantial synergy in KRAS-mutated NSCLC in vivo [191].

#### 6.3.3. Other CDKIs Combined with Radiotherapy

Recently, an in vitro study by Tai et al. demonstrated that the selective CDK4/6 inhibitor ribociclib, also known as LEE011, induced cell-cycle arrest in the OSCC cell lines SCC4 and SCC25 cells during the G1/M phase through inhibition of Rb phosphorylation. Moreover, the effects of radiation were enhanced in HNSCC cell line OML1 and its radioresistant clone OML1-R. It was easier to overcome radioresistance with additional use of the CDK4/6 inhibitor flavopiridol [192]. Jung et al. screened 14,600 compounds for their ability to sensitize tumor cells for radiation. They discovered the small molecule radiosensitizer PPA15. It was found to be a pan-CDKI which, combined with radiation, resulted in suppression of A549 (lung adenocarcinoma) tumor growth in mice by 50–60% [193].

Kodym et al. investigated the effect of the small peptide SNS-032, a selective CDK2/7/9 inhibitor, on radiosensitivity of NSCLC. This CDKI acts independently of the cell cycle. This provides a tremendous advantage in the treatment of silent, non/low proliferating tumor subpopulations, which are a major cause of radioresistant relapse. Additionally, in non-cyclic cells, a delay in the resolution of radiation-induced gammaH2AX foci, mediated by SNS-032, was detected. These foci are a surrogate for DNA double-strand breaks (DSB), suggesting modulation of DNA DSB repair [194].

Furthermore, inhibition of CDK1, CDK2 and CDK9 in NSCLC by AZD5438 resulted in a dose-dependent increase in radiosensitivity. Notably, the degree of radiosensitization by AZD5438, a new-generation inhibitor, was stronger in radioresistant cell lines. The underlying mechanism is that the small molecule radiosensitizer PPA15 is responsible for inhibition of CDK1, prolonged G2/M phase arrest, inhibition of HR, delayed DNA DSB repair and increased apoptosis. [195].

In HNSCC, modulation of CDK9 expression led to alterations in radiosensitivity. While overexpression of CDK9 mediated radioprotection in five stably CDK9-EGFP-N1 transfected HNSCC cell lines. Depletion of CDK9 by siRNA knockdown clearly enhanced the radiosensitivity of HNSCC cells without an induction of apoptosis [196].

### 6.4. Influence of CDKIs on Chemosensitization/Targeted Therapy

There are also several studies on the effect of combination therapy with CDK4/6 inhibitors in HNSCC. Several studies are currently evaluating palbociclib monotherapy as well as palbociclib treatment in combination with chemotherapy (cisplatin, carboplatin), targeted therapy (cetuximab, gedatolisib), immunotherapy (avelumab) or radiotherapy.

In HNSCC and other tumor entities, it was observed that EGFR-signaling was dominant, but targeted antibody therapy resulted in an extremely low clinical response. In most cases, intrinsic resistance mechanisms and activation of alternative signaling pathways are responsible for this outcome. Several studies have successfully shown a synergistic effect of CDK4/6 inhibition when combined with other inhibitors targeting the AKT, MAPK/ERK or mTOR1 pathway in various types of cancer [197,198,199]. Therefore, combined administration of a CDK4/6 inhibitor with chemo- or targeted therapies may be a hopeful treatment against various malignancies.

To show only a few examples: under hypoxia, Palbociclib synergizes with irinotecan, a cytostatic drug for the monotherapy of 5-FU-resistant, advanced, metastatic colon carcinoma, to promote colorectal cancer cell death [200]. Synergy of palbociclib with a MEK inhibitor has also been demonstrated in KRAS mutant colon cancer in vivo [197,201]. Vemurafenib-resistant tumors remain sensitive to palbociclib, suggesting that initial treatment with vemurafenib, a selective inhibitor of the oncogene BRAF, followed by palbociclib with or without mTOR inhibitors may be an effective therapeutic approach to manage relapse of vemurafenib-resistant metastatic tumors [202]. In a Phase I-II open-label multicenter study of palbociclib and vemurafenib, patients with unresectable stage III or stage IV BRAF^V600MUT^ metastatic melanoma were treated with palbociclib once daily for 14 days followed by a 7-day break and continuous dosing of vemurafenib. Here, a significant clinical benefit was achieved in pretreated patients with melanoma [203].

The first phase I clinical trial in recurrent and metastatic HNSCC combining palbociclib and cetuximab was performed in 2016. It was found that the combination therapy posed no additional risk to patient safety [139]. A multicenter, multigroup Phase 2 trial with palbociclib and cetuximab in platinum-resistant and in cetuximab-resistant HPV-negative HNSCC was conducted in 2019.

This study reports that palbociclib in combination with cetuximab exhibited substantial antitumor activity in a platinum-resistant group (group 1) and in a cetuximab-resistant group (group 2). The proportion of patients achieving an objective response with palbociclib and cetuximab was 39% in group 1 and 19% in group 2 [204]. In the PALATINUS phase II trial among patients with platinum-resistant, HPV-unrelated HNSCC, palbociclib plus cetuximab resulted in a trend of prolongation of median overall survival (OS) compared with cetuximab. The median OS was 9.7 months in the palbociclib arm and 7.8 months in the placebo arm [205].

In tumor xenografts, Robinson et al. reported palbociclib to be highly effective against chemo naive HNSCC cell lines. However, prior cisplatin exposure induced resistance to palbociclib. The reason for this could be an upregulation of c-Myc and cyclin E after cisplatin exposure. Combination treatment with palbociclib and the c-Myc bromodomain inhibitor JQ1 exerted a synergistic anti-growth effect in cisplatin-resistant cells [206]. In HNSCC cell lines, Beck et al. demonstrated that afatinib or lapatinib, both tyrosine kinase inhibitors, combined with palbociclib had promising activity in terms of inhibition of tumor cell viability. However, in this study, the combination was not investigated in in vivo preclinical models [207].

A recent study by Chaudhary et al. demonstrated afatinib and palbociclib in combination decreased the proliferation of HNSCC cells by inducing cell cycle arrest. It was also shown to induce ROS production and senescence [186].

Gadsen et al. found that CDK4/6 inhibitors lead to senescence in HPV-negative HNSCC, but not in HPV-positive HNSCC. The BCL-2 family inhibitor, navitoclax, has been shown to eliminate senescent cells effectively. They also found that combining palbociclib with navitoclax led to decreased HPV-negative HNSCC cell survival and led to increased apoptosis levels in HPV-negative cell lines compared with each agent given alone [208].

In a xenograft mouse model of HNSCC, palbociclib and trametinib, an inhibitor of MEK/ERK pathway, synergistically inhibited tumor growth and enhanced tumor cell apoptosis [209].

Zainal et al. demonstrated that palbociclib resistant OSCC cells harbored PIK3CA mutations and were less responsive to palbociclib compared to wild-type cells. They found out that combination treatment with a PI3K/mTOR inhibitor and palbociclib completely controlled tumor growth in mice [210].

In some cases, inhibition of CDKs has been found to abrogate mechanisms which enable aberrant cells to escape apoptosis. This in turn led to an increase in TNF-related apoptosis-inducing ligand (TRAIL) sensitivity via activation of caspase-8. [211]. Chen et al. demonstrated that the use of flavopiridol led to the upregulation of several BH3-only proteins such as BimEL, Noxa, and Bik/NBK. This allowed them to sensitize human myeloma cells to BH3-mimetic drugs. BH3 mimetics induce apoptosis by antagonizing the activity of anti-apoptotic Bcl-2 family proteins [212].

In hypopharyngeal carcinoma, CDKI-73, a potent CDK9 inhibitor, was shown to suppress CDK 9 mediated phosphorylation of RNA polymerase II and thus triggered downregulation of Mcl-1 expression. Mcl-1 together with Bcl-2 is, in its full length, an antiapoptotic protein [213]. CDKI-73 was also able to potentiate the induction of apoptosis in combination with cisplatin in hypopharyngeal carcinoma [214].

Additionally, Syn et al. reported a synergistic effect of roniciclib (BAY1000394), a potent pan-CDKI, and cisplatin in preclinical nasopharyngeal carcinoma (NPC) models. They showed that blockage of cell cycle CDK1, CDK2, CDK3, and CDK4 and transcriptional CDK7 and CDK9, resulted in G1/S and G2/M arrest and repression of anti-apoptotic proteins. This effect occurred only in transformed neural progenitor cells but not in non-tumorigenic nasopharyngeal epithelial cell line NP69. Moreover, combination of roniciclib and cisplatin in BALB/c xenograft mice led to significantly increased tumor suppression compared to monotherapies [215].

Although experimental data seem promising, there are currently data from one clinical trial in HNSCC combining palbociclib with carboplatin, in which there is no clear benefit from the combination but increase toxicity [216]. However, there is evidence of benefit from those combination in other tumor types, in which the order of administration seems to be critical for CDK4/6 inhibition potentiating the antiproliferative effect of genotoxic therapies [217].

### 6.5. Influence of CDKIs on Immunotherapy

Tumor cell adaption and immune evasion may occur due to a variety of molecular dysregulations. A common mechanism is the alteration of molecules that cause immune evasion by malfunction of the antigen-presenting machinery via class I HLA molecules. Another mechanism to evade the immune system may be the reintroduction of cyclin D/CDK4 heterodimers into cell cycle.

Recent research identified that reintroduction of cyclin D/CDK4 in the chromatin assembly complex (CAF), responsible for the reposition of histones H3 and H4 at the DNA replication fork, would destabilize PD-L1, a negative regulator of the immune checkpoint PD-1, via the cullin 3-SPOP pathway [218]. The expression of PD-L1 by tumor cells is mainly associated with its immunosuppressive effect. In fact, PD-1/PD-L1 immune checkpoint inhibitors demonstrated remarkable effects in advanced cancer patients, including HNSCC [219,220,221]. Loss of function mutations of SPOP, on the other hand, increase the level of PD-L1, thereby reducing the presence of tumor-infiltrating lymphocytes in the tumor environment. This has been observed in mouse models. CDK4/6 inhibitors could, theoretically, increase PD-L1 levels, thereby potentiating the immune checkpoint blockade (ICB) treatment [222].

**Figure 19:**
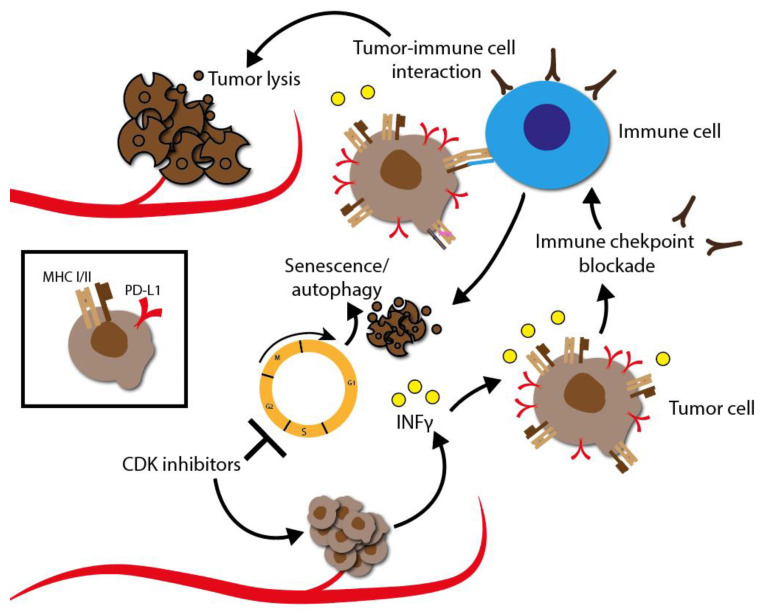
Sensitization to immunotherapy by CDKIs. CDK inhibition prevents cell cycle progression and triggers an IFNγ response accompanied by MHC I/II upregulation. CDK inhibition also leads to stabilization of PD-L1 membrane expression of tumor cells. The additional use of immune checkpoint inhibitors, such nivolumab and pembrolizumab, blocks the interaction between the inhibitory checkpoint PD-1 and its ligand PD-L1. This prevents resistance to the immune response. As a result, cytotoxic T cells proliferate, leading to efficient tumor cell killing and clinical response.

Sensitizing tumor cells to chemotherapy or radiotherapy and simultaneously stimulating the immune system with CDKI is promising to prevent or counteract resistance mechanisms. CDKIs can be extremely helpful in activating specific targets that are important for the development of resistance. Therefore, it is hoped that treatment of patients with CDK inhibitors, especially in combination with immune checkpoint inhibitors, will improve overall response. Figure 19 schematically describes how the mechanism could look like.

Currently, there are five clinical trials investigating the combined efficacy of CDKIs and immunotherapy (NCT03938337, NCT04000529, NCT03498378, NCT03655444, NCT04213404) in HNSCC. Crucial seem the relevant adverse effects, particularly neutropenia as well as rise of liver enzymes, renal disorders and pulmonary toxicity which led to the termination of two studies (NCT03938337, NCT03655444).

## 7. Summary and Conclusions

Considering the tremendous role of CDKs during cell division, it is not surprising that tumor cells have developed a number of strategies to disable or bypass these key components [8]. Cell division is essential for the development and maintenance of a healthy organism.

Each phase transition in the cell cycle is regulated by a specific subset of cyclins and CDKs. CDKs are involved as key regulatory enzymes that sensitively control all cell cycle transitions (Figure 2). The action of CDKs is controlled by specific activation and inactivation by other proteins. Typically, cyclin/CDK complexes are activated by phosphorylation of the CDK-activating kinase. On the contrary, these complexes can be negatively regulated by several cell intrinsic CDKIs. Based on their structural and functional properties, cell intrinsic CDKIs belong to two large families, INK4 and Cip/Kip. The cell intrinsic CDK inhibition mechanism involves interaction between the p16- and CDK6-charged binding domains, resulting in reduced kinase activity and a decreased cyclin-binding surface.

Since cell cycle dysregulation may play a key role in tumor development, many different CDKIs have been developed as anti-cancer drugs.

The early developed CDKIs, like flavopiridol and roscovitine, met only moderate clinical success with partly severe side effects. It was later concluded that the main reason for their failure was the functional diversity of individual CDKs. The use of non-specific CDK pan-inhibitors that addressed several CDKs simultaneously triggered uncontrollable effects which could ultimately cause more harm than good in cancer patients. The focus of further development was therefore on increasing the selectivity of CDKIs. Next generation CDKIs were drugs like dinaciclib and AT7519 as well as small and stapled peptides. Although non-specific pan-CDKIs have shown disappointing clinical results in a broad range of applications, there is nevertheless some evidence that non-specific pan-CDKIs are also actually effective in a limited range of applications. Interestingly, recent studies have shown that combination therapies, in contrast to CDKI monotherapies, can provide promising results in tumor therapy.

Due to their ubiquitous biological functions, many CDKs are of limited use as clinical targets. Because of the narrow therapeutic window, specific CDKIs are necessary to provide a promising treatment with reduced side effects. A variety of clinical studies on specific CDKIs, targeting for example CDK1, CDK2, CDK7, CKD8/19, CDK9 and CDK12/13 are currently in progress. The development of selective inhibitors, of both CDK4 and CDK6, has markedly changed the perception of CDKs as therapeutic targets in cancer. Dual CDK4 and CDK6 inhibitors have been shown to be active in multiple preclinical models, including xenografts, genetically engineered mouse models and primary human tumor explants. The specific CDK4/6 inhibitors albociclib, ribociclib and abemaciclib, are the most studies ones. Unfortunately, approximately 10% of patients will have primary resistance to CDK4/6 inhibitors.

Although CDKI monotherapy results in partly quite disappointing outcomes, combination therapies appear to have some advantages in tumor therapy. The advantages of combining CDKIs together with standard therapies, such as radio-, chemo-, targeted or even immunotherapy, in various tumor types showed a clear tendency towards a synergistic effect of CDKIs, which may ultimately lead to the sensitization of radio- and chemoresistance as well as a sensitization to immune checkpoint blockade. The synergistic effects that contribute overcoming resistance have been observed in several experimental studies and early clinical trials on HNSCC, NSCLC and pancreatic carcinoma, for instance. Especially in tumor entities such as HNSCC, where the 5-year overall survival rate of patients is still around 50% despite remarkable progress in the last decades, such findings are enormously important. The potential that combination therapies offer, led, with good reason, to a veritable renaissance of CDKIs.

## Figures and Tables

**Table 1:** Collection of some CDK degrading PROTACs currently under development.

PROTAC	Target	Reference
Prodrug 11	CDK2/4/6	[48]
Pal-pom	CDK4/6	[49]
BSJ-03-123	CDK4/6	[50]
PROTAC 6	CDK6	[51]
CP-10	CDK6	[52]
YX-2-107	CDK6	[53]
CST651	CDK6	[54]
JH-XI-10-02	CDK8	[55]
PROTAC 3	CDK9	[56]
PROTAC 11c	CDK9	[57]
B03	CDK9	[58]
THAL-SNS-032	CDK9	[59]
F3	CDK9/CDK2	[60]

**Table 2:** Pan-CDK inhibitors evaluated in clinical phase trials.

Drug	Synonym	IC_50_	Target	Progress	Application
Flavopiridol	Alvocidib, L868275, HMR-1275	20–100 nM	CDK1,2,4,6,7,9	Phase II NCT03604783	ALL, AML, CLL, MM, lymphoma, MCL
Roscovitine	CYC202, Seliciclib	0.16–0.7 µM	CDK1,2,5,7,9	Phase II NCT03774446	NSCLC, Crohn’s disease, Niemann Pick Disease Type C, metastatic breast cancer, advanced solid tumor
Dinaciclib	SCH 727965, SCH-727965	1–4 nM	CDK1,2,5,9	Phase III NCT01580228	CLL, MCL, NSCLC, melanoma, breast cancer
P276-00	Riviciclib hydrochloride, P276	20–79 nM	CDK1,4,9	Phase II NCT00899054	BC, HNSCC
TG02	SB1317, TG-02, SB-1317	n.a.	CDK1,2,5,7,9	Phase II NCT03904628	glioblastoma, anaplastic astrocytoma, CLL, hematological neoplasm
AT7519	AT 7519, AT-7519	10–210 nM	CDK1,2,4,5,6,9	Phase II NCT02503709	MM, CLL, MCL, NHL, solid tumors
Roniciclib	BAY1000394	1–25 nM	CDK1,2,3,4,7,9	Phase II NCT02161419	SCLC
RGB-286638	RGB286638	1–5 nM	CDK1,2,3,4,5,9	Phase I NCT01168882	hematological malignancies
PHA-793887	PHA 793887, PHA793887	5–10 nM	CDK1,2,4,5,7,9	Phase I NCT00996255	solid tumors
SNS032	BMS-387032, SNS-032	48–62 nM	CDK1,2,4,7,9	Phase I NCT00446342	B-lymphoid malignancies, CLL, solid tumors
R547	Ro 4584820	2–3 nM	CDK1,2,3,4,7,9	Phase I NCT00400296	neoplasms
Indirubin	Isoindigotin, Indigopurpurin	0.8–1 µM	CDK1,2,4,5	Phase IV NCT02200978	childhood acute promyelocytic leukemia
AZD-5438	AZD5438; AZD 5438	6–20 nM	CDK1,2,4,5,7,9	Phase I NCT00088790	neoplasms
CYC065	CYC-065, CYC 065	5/26 nM	CDK2,9	Phase I NCT02552953	AML, MDS, Advanced cancer, Relapsed/ Refractory CLL
AG024322	AG-024322	120 nM	CDK1,2,4,6,7,9	Phase I NCT00147485	neoplasms, Non-Hodgkin lymphoma
Voruciclib	P1446A-05	22–90 nM	CDK4,6,9	Phase I NCT03547115	CLL/melanoma

**Table 3:** Overview of selected specific CDK inhibitors.

Compound	IC_50_	Target	Progress	Number of Trials	Application	Literature
Sorafenib	6 nM	CDK1	Phase IV NCT02733809, NCT02504983, NCT03518502	3	hepatocellular carcinoma, fibrolamellar, leukemia, thyroid	[92]
RO3306	20–1980 nM	CDK1	n.a.	n.a.	n.a.	[93]
HI 5	6 µM	CDK2	Phase III NCT01566695 NCT04143451 NCT04266301	6	Leukemia, renal carcinoma	[94]
Palbociclib	11/16 nM	CDK4/6	Phase IV	2	breast cancer	[95]
Ribociclib	10/39 nM	CDK4/6	NCT03355157, NCT03220178	10	breast cancer	[96]
Abemaciclib	2/10 nM	CDK4/6	Phase III	1	breast cancer	[97]
Trilaciclib	1/4 nM	CDK4/6	NCT02422615	1	SCLC	[98]
SHR6390	12 nM/10µM	CDK4/6	NCT01958021	3	breast cancer	[99]
G1T38/Lerociclib	1 nM/2nM	CDK4/6	Phase II NCT02983071, NCT03455829	2	breast cancer, NSCLC	[100]
XZP-3287/Birociclib	n.a.	CDK4/6	Phase II NCT04539496	1	breast cancer, solid tumors	[101]
SY5609	<6 nM	CDK7	Phase I NCT04247126, NCT04929223	2	breast, NSCLC, colorectal,	[102]
XL102	n.a.	CDK7	Phase I NCT04726332	1	breast, ovarian, prostate	n.a.
LDC3140, LDC4297, THZ1, THZ2	0.13 nM	CDK7	n.a.	n.a.	n.a	[103]
RVU120	4.4/10.4 nM	CDK8/19	Phase I NCT04021368	1	acute Myeloid Leukemia	n.a.
cortistatin A, CCT251545, CCT251921, MSC2530818	100 nM	CDK8/19	n.a.	n.a.	n.a.	[104,105,106,107,108,109]
AZD4573	<3 nM	CDK9	Phase II NCT04630756	1	advanced hematological malignancies	
TP-1287	n.a.	CDK9	Phase I NCT03604783	1	solid tumors, sarcoma	[110]
GFH009	n.a.	CDK9	Phase I NCT04588922	1	hematologic malignancies, AML, CML, SLL, lymphoma	[111]
KB-0742	6 nM	CDK9	Phase I NCT04718675	1	solid tumors, Non-Hodgkins Lymphoma	[112]
Fadraciclib	26 nM	CDK9	Phase II NCT04983810, NCT05168904	2	solid tumor, leukemia, lymphoma	[113]
SR-4835/THZ531	4.9 nM	CDK12/13	n.a.	n.a.	n.a.	[114]

**Table 4:** Ongoing studies of CDK 4/6 therapies in HNSCC (from clinicaltrials.gov, 2021).

Study Type	Study Status	NCT Number	Tumor Entity	Title/ Intervention	Treatment Schedule	Primary Outcome	Results
Open-label, single arm, Phase II	Recruiting	03356223	HNSCC	Abemaciclib monotherapy for locally advanced/ metastatic HNSCC after failure of platinum and cetuximab or anti-EGFR-based therapy and harboring homozygous deletion of CDKN2A, and/or amplification of CCND1 and/or CDK6	Abemaciclib: 400 mg/day two doses of 200 mg 12-h apart. For each 28-day cycle, a total of 56 doses of study drug will be dispensed	8-week non-progression rate defined as the rate of patients with complete response (CR), partial response (PR) or stable disease (SD) lasting at least 8 weeks	Ongoing
Interventional, phase I/ II, dose escalation	Recruiting	03024489	HNSCC	Albociclib in combined with cetuximab and Intensity Modulated Radiation Therapy (IMRT) for locally advanced HNSCC	Palbociclib: 100 mg oral every day 3 week-on and 1-week off during IMRT Cetuximab: 400 mg/m2 IV at 7 days before (day -7) starting radiation. Then 250 mg/m2 IV weekly for 7 weeks. IMRT: 5 days on/2 days off with a total dose of 70 Gy for 33–35 fractions	Determination of dose-limiting toxicities (DLTs) and recommended phase II dose (RP2D)	Ongoing
Interventional, open-label, Phase I, single arm	Recruiting	03065062	Lung cancer squamous cell, solid tumors, HNSCC, pancreatic cancer	Palbociclib in combination with PI3K/mTOR inhibitor pedatolisib (PF-05212384) for patients with advanced squamous cell lung, pancreatic, HNSCC and other solid tumors	Palbociclib: orally, once daily, 3 weeks out of every 4 in each cycle. Initial dose for part 1 of the study 100 mg daily. Gedatolisib: once weekly on the first day for each of the 4 weeks during the 4-week cycle. Initial dose for part 1 of the study 110 mg	Maximum Tolerated Dose and Recommended Phase II dose Incidence of Treatment-Emergent Adverse Events (AE)	Ongoing
Interventional, Phase I, open label, parallel assignment	Recruiting	02897375	Solid neoplasms incl. HNSCC	Palbociclib in combination with cisplatin or carboplatin in advanced solid malignancies	Cisplatin: IV over 30–60 min on day 1 Palbociclib: PO QD on days 2–22 or: Carboplatin: IV over 30–60 min on day 1 and Palbociclib: PO QD on days 2–22	Safety and tolerability of palbociclib when administered along with cisplatin or carboplatin. Recommended phase II dose (RP2D) of the tested combinations	Ongoing
Interventional, phase II, single group, open label	Recruiting	04169074	HNSCC	Immune modulation by abemaciclib in HPV-negative HNSCC	Abemaciclib: from days 1–21 in both arms. May be continued for additional 7 days, or up to 28 days, for delays in planned surgery	Measure quantitative change in tumor size to assess the clinical activity of abemaciclib	Ongoing
Interventional, phase I/II, non-randomized, sequential assignment, open label	Terminated (Provider of drug decided to discontinue study)	03655444	HNSCC	Abemaciclib in combination with Nivolumab in Patients with R/M HNSCC, progressed or recurred within 6 months after platinum-based chemotherapy	Abemaciclib: (150 mg) orally twice per day on Days 1 through 28 of every 4-week cycle, Nivolumab: 480 mg IV over 30 min on day 1 of every 4-week cycle	Determination of recommended Phase 2 dose of Abemaciclib combined with fixed dose of Nivolumab, Overall Survival, Best Overall Tumor Response, duration of Tumor Response	Study terminated, n = 6, recommended dose of Abemaciclib: 150 mg twice a day, OS 3.7 months
Interventional, phase I, single group assignment, open label	Active, not recruiting	04213404	HNSCC	Ribociclib in combination with Spartalizumab in R/M HNSCC (RISE-HN)	Ribociclib: 400 mg, 600 mg, or 200 mg oral daily, day 1–21, 28 days a cycle, Spartalizumab: 400 mg IV on day 1, 28 days a cycle	Progression free survival, Overall survival, Duration of response, Objective response rate	Ongoing

## Abbreviations

ALL	acute lymphoblastic leukemia
AML	acute myeloid leukemia
Antp	Drosophila Antennapedia protein
BCL	B cell lymphoma
BRCA	Breast Cancer-associated Protein
CAF	chromatin assambly complex
CAK	CDK-activating kinase
CDK	cyclin-dependent kinase
CDKI	cyclin-dependent kinase inhibitor
Cip/Kip	CDK interacting protein/Kinase inhibitory protein
CLL	chronic lymphocytic leukemia
CPP	cell penetrating peptide
CR	complete response
CRC	colorectal cancer
DLT	dose-limiting toxicity
DSB	double-strand breaks
EGFR	epidermal growth factor receptor
EMT	epithelial-mesenchymal transition
HCC	hepatocellular carcinoma
HNSCC	head and neck squamous cell carcinoma
HPV	human papillomavirus
IC_50_	half maximal tolerated dose
ICB	immune checkpoint blockade
IFN	Interferon
LCSC	lung cancer squamous cell
M phase	mitosis phase
MCL	mantle cell lymphoma
MM	multiple myeloma
MTD	maximum tolerated dose
NHL	non-Hodgkin’s lymphoma
NPC	nasopharyngeal carcinoma
NSCLC	non-small cell lung cancer
OS	overall survival
OSCC	oral squamous cell carcinoma
PDK1	3-phosphoinositide-dependent protein kinase 1
PDX	patient derived xenograft
PFS	Progression-Free Survival
PPI	protein-protein interactions
PR	partial response
pRb	phosphorylated retinoblastoma protein
PROTAC	proteolysis targeting chimera
P-TEFb	positive transcription elongation factor b
Rb	retinoblastoma protein
RP2D	recommended phase II dose
R-point	restriction point
RNA Pol II	RNA Polymerase II
S phase	synthesis phase
SCLC	Small-cell lung cancer
SLL	small lymphocytic lymphoma
Ser/Thr	serine/threonine
SR	stable disease
T-ALL	T cell acute lymphoblastic leukemia
TGF	Transforming Growth Factor
TK1	Thymidine Kinase 1
TRAIL	TNF-related apoptosis-inducing ligand
